# Supplementary figures and images for: What’s all that racket! Soundscapes, phenology, and biodiversity in estuaries
Source: PLoS One. 2020 Sep 3;15(9):e0236874. doi: 10.1371/journal.pone.0236874 (PMC7470342; doi:10.1371/journal.pone.0236874)

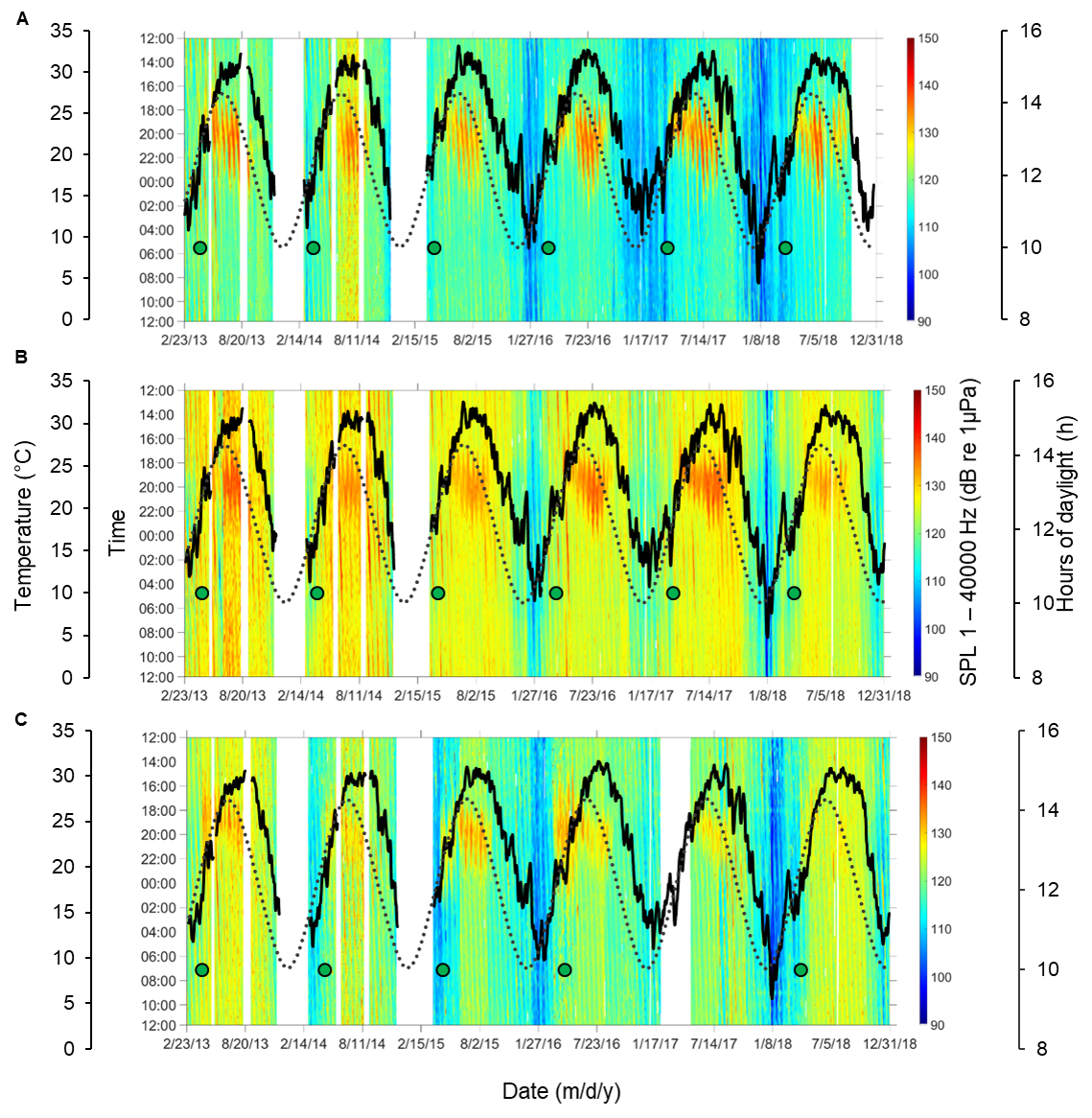

Supplement: S1 Fig — Heat maps represent temporal and spatial patterns of broadband (i.e. 1–40,000 Hz) frequency SPLs reflecting all physical sounds, biological sounds, and anthropogenic noise at stations (A) 9M, (B) 14M, and (C) 37M in the May River. Time is shown between noon and noon of the next day. Gaps in data = white, temperature = black line, and daylight hours = dotted line. Green dots indicate first posterior probability (PP) ⩾ 0.5 detected during springtime. At station (C) 37M first PP was not calculated for spring 2017 due to missing acoustic data. (TIF) [file pone.0236874.s001.tif]

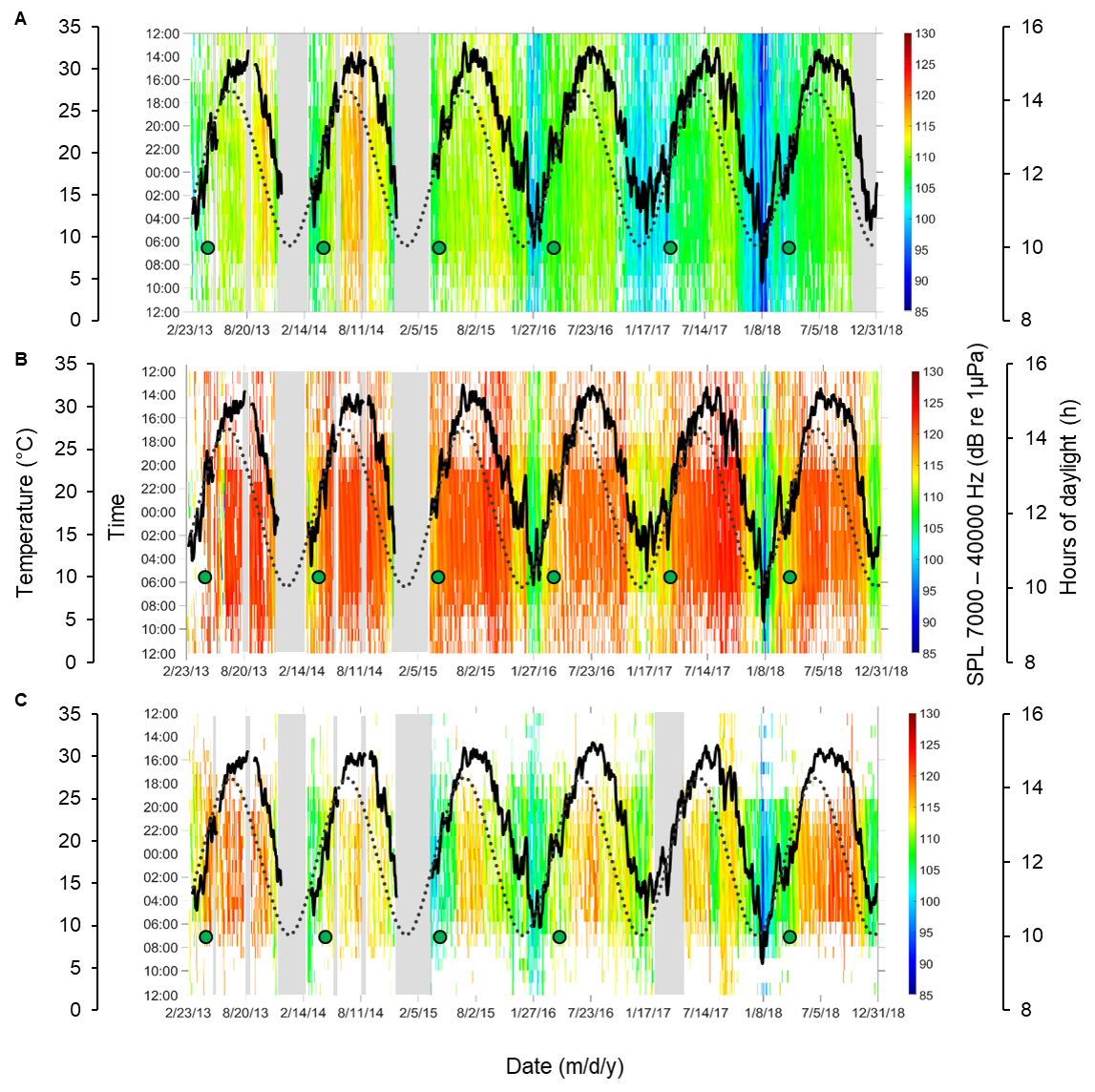

Supplement: S2 Fig — Heat maps represent temporal and spatial patterns of high (i.e. 7000–40,000 Hz) frequency SPLs reflecting snapping shrimp acoustic activity at stations (A) 9M, (B) 14M, and (C) 37M in the May River. Time is shown between noon and noon of the next day. Gaps in data = gray, files with physical sounds and anthropogenic noise removed = white, temperature = black line, and daylight hours = dotted line. Green dots indicate first posterior probability (PP) ⩾ 0.5 detected during springtime. At station (C) 37M first PP was not calculated for spring 2017 due to missing acoustic data. (TIF) [file pone.0236874.s002.tif]

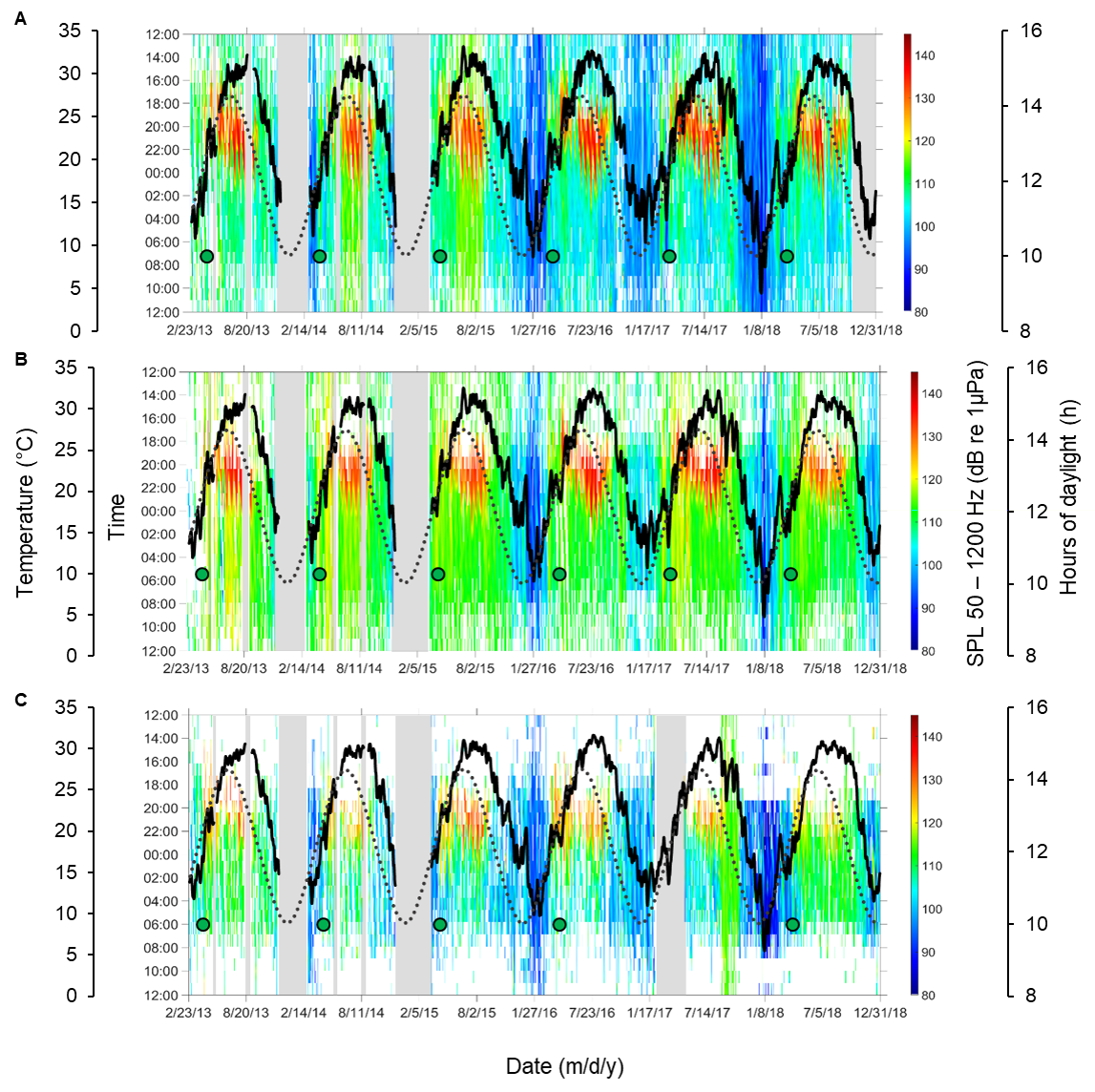

Supplement: S3 Fig — Heat maps represent temporal and spatial patterns of low (i.e. 50–1200 Hz) frequency SPLs reflecting fish and lower portion of snapping shrimp acoustic activity at stations (A) 9M, (B) 14M, and (C) 37M in the May River. Time is shown between noon and noon of the next day. Gaps in data = gray, files with physical sounds and anthropogenic noise removed = white, temperature = black line, and daylight hours = dotted line. Green dots indicate first posterior probability (PP) ⩾ 0.5 detected during springtime. At station (C) 37M first PP was not calculated for spring 2017 due to missing acoustic data. (TIF) [file pone.0236874.s003.tif]

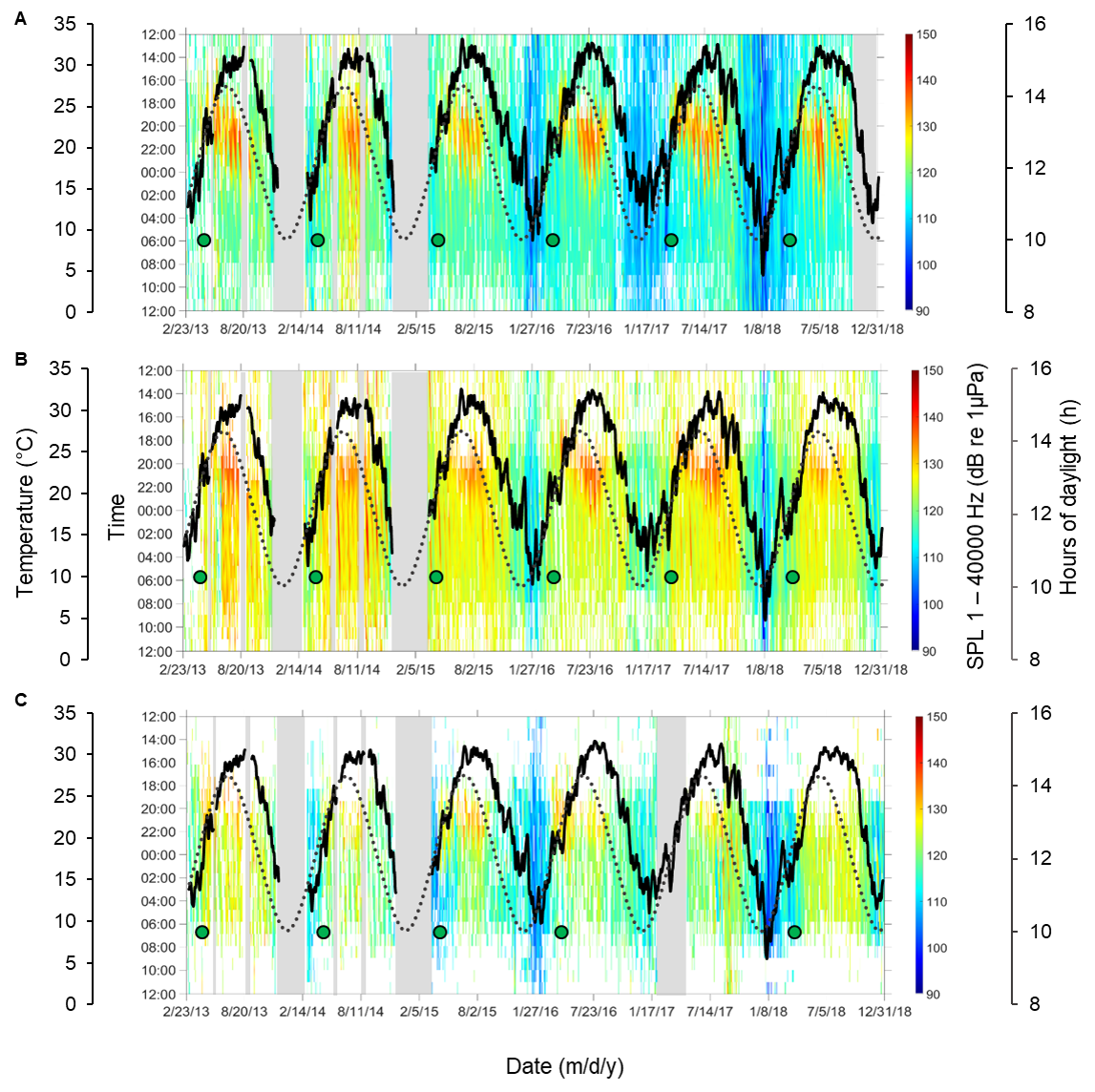

Supplement: S4 Fig — Heat maps represent temporal and spatial patterns of broadband (i.e. 1–40,000 Hz) frequency SPLs reflecting all biological activity at stations (A) 9M, (B) 14M, and (C) 37M in the May River. Time is shown between noon and noon of the next day. Gaps in data = gray, files with physical sounds and anthropogenic noise removed = white, temperature = black line, and daylight hours = dotted line. Green dots indicate first posterior probability (PP) ⩾ 0.5 detected during springtime. At station (C) 37M first PP was not calculated for spring 2017 due to missing acoustic data. (TIF) [file pone.0236874.s004.tif]

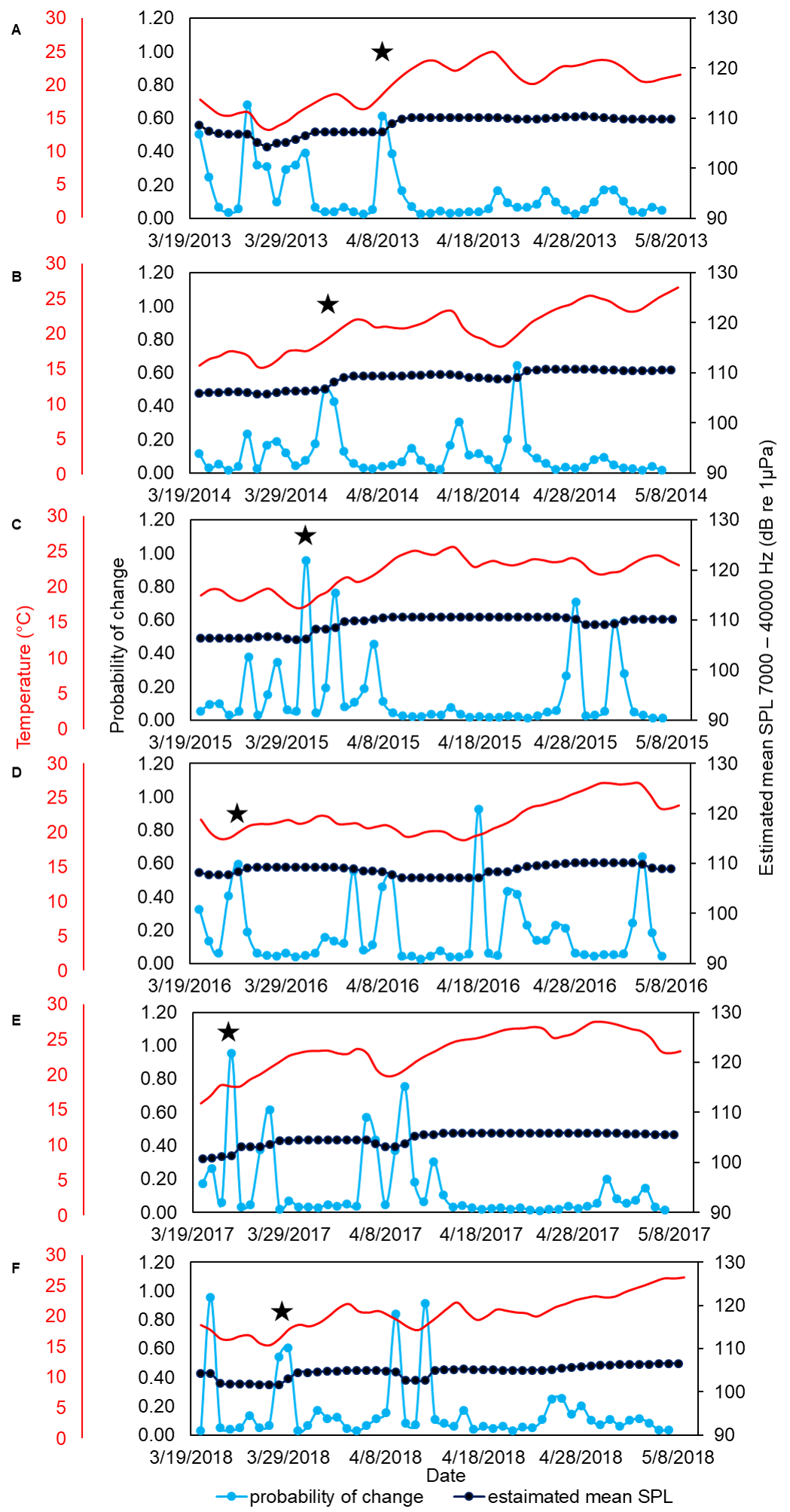

Supplement: S5 Fig — Posterior probability of change during springtime (light blue line) with corresponding estimated mean high (7000–40,000 Hz) frequency SPL (dark blue line) and corresponding water temperature (red line) in years (A) 2013, (B) 2014, (C) 2015, (D) 2016, (E) 2017, and (F) 2018. Stars indicate first positive (i.e. detected change in estimated mean SPL due to an increase not a decrease in SPL values) PP ⩾ 0.5. (TIF) [file pone.0236874.s005.tif]

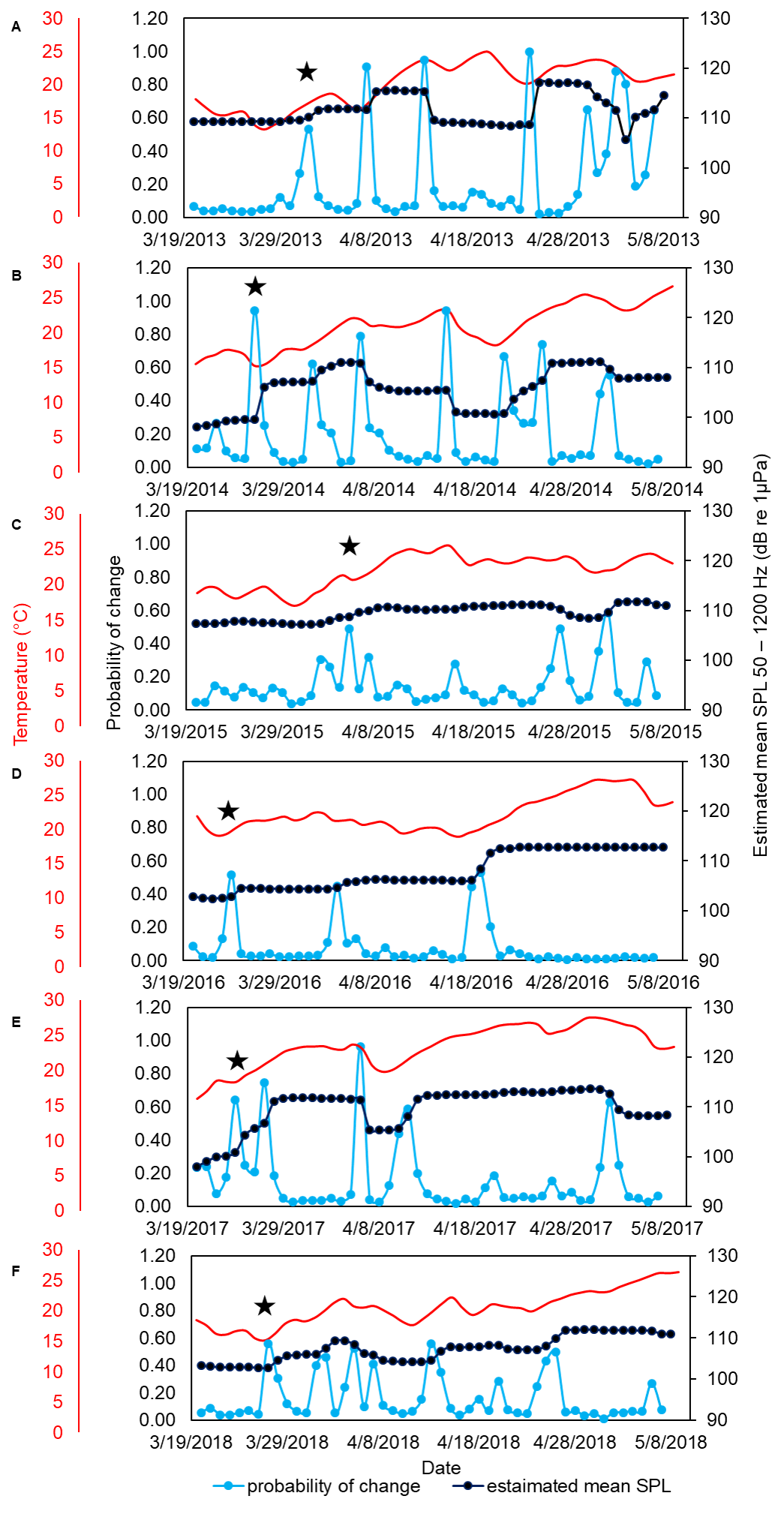

Supplement: S6 Fig — Probability of change during springtime (light blue line) with corresponding estimated mean low (50–1200 Hz) frequency SPL (dark blue line) and corresponding water temperature (red line) in years (A) 2013, (B) 2014, (C) 2015, (D) 2016, (E) 2017, and (F) 2018. Stars indicate first positive (i.e. detected change in estimated mean SPL due to an increase not a decrease in SPL values) PP ⩾ 0.5. (TIF) [file pone.0236874.s006.tif]

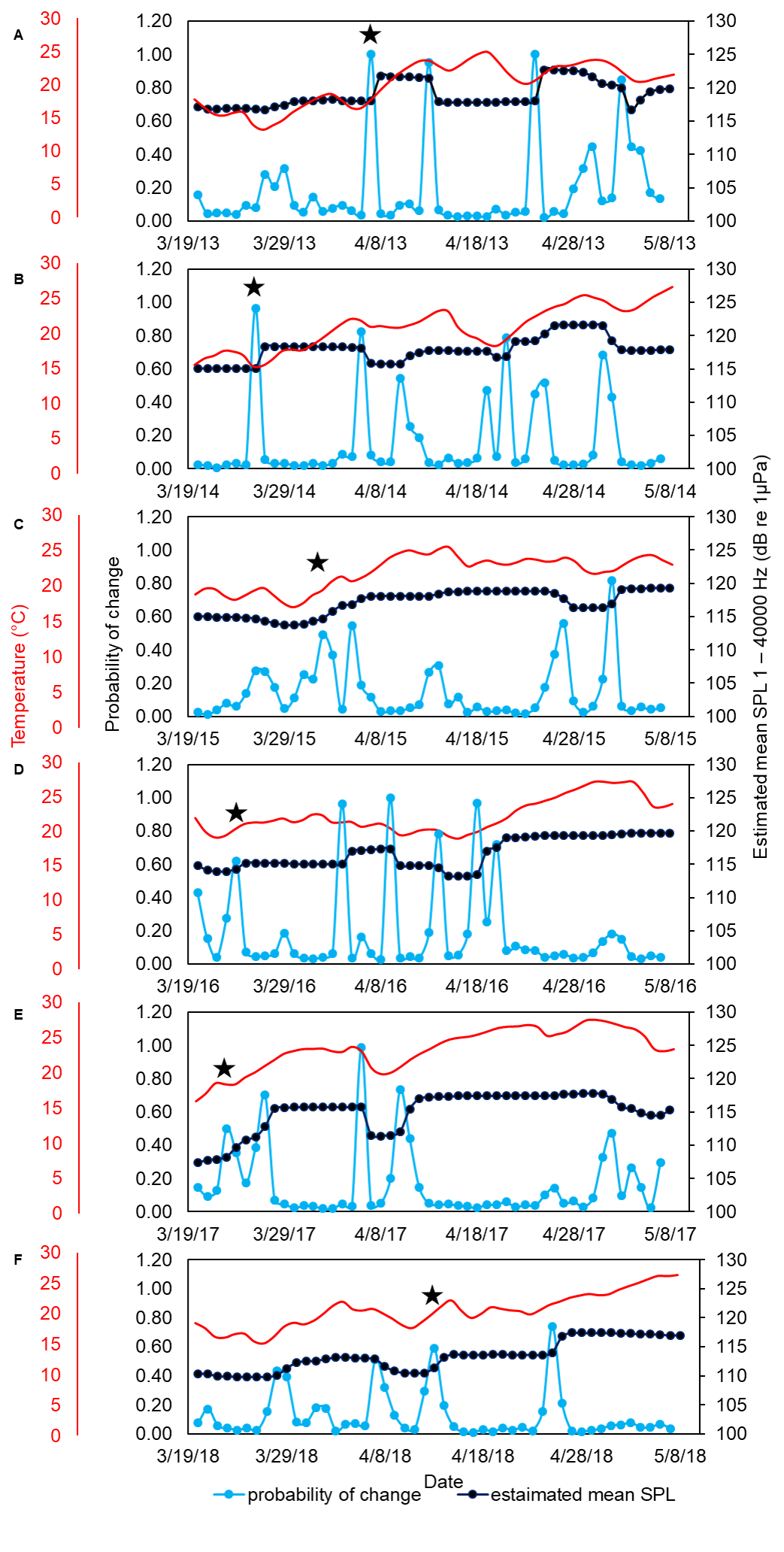

Supplement: S7 Fig — Probability of change during springtime (dark blue line) with corresponding estimated mean broadband (1–40,000 Hz) frequency SPL (light blue line) and corresponding water temperature (red line) in years (A) 2013, (B) 2014, (C) 2015, (D) 2016, (E) 2017, and (F) 2018. Stars indicate first positive (i.e. detected change in estimated mean SPL due to an increase not a decrease in SPL values) PP ⩾ 0.5. (TIF) [file pone.0236874.s007.tif]

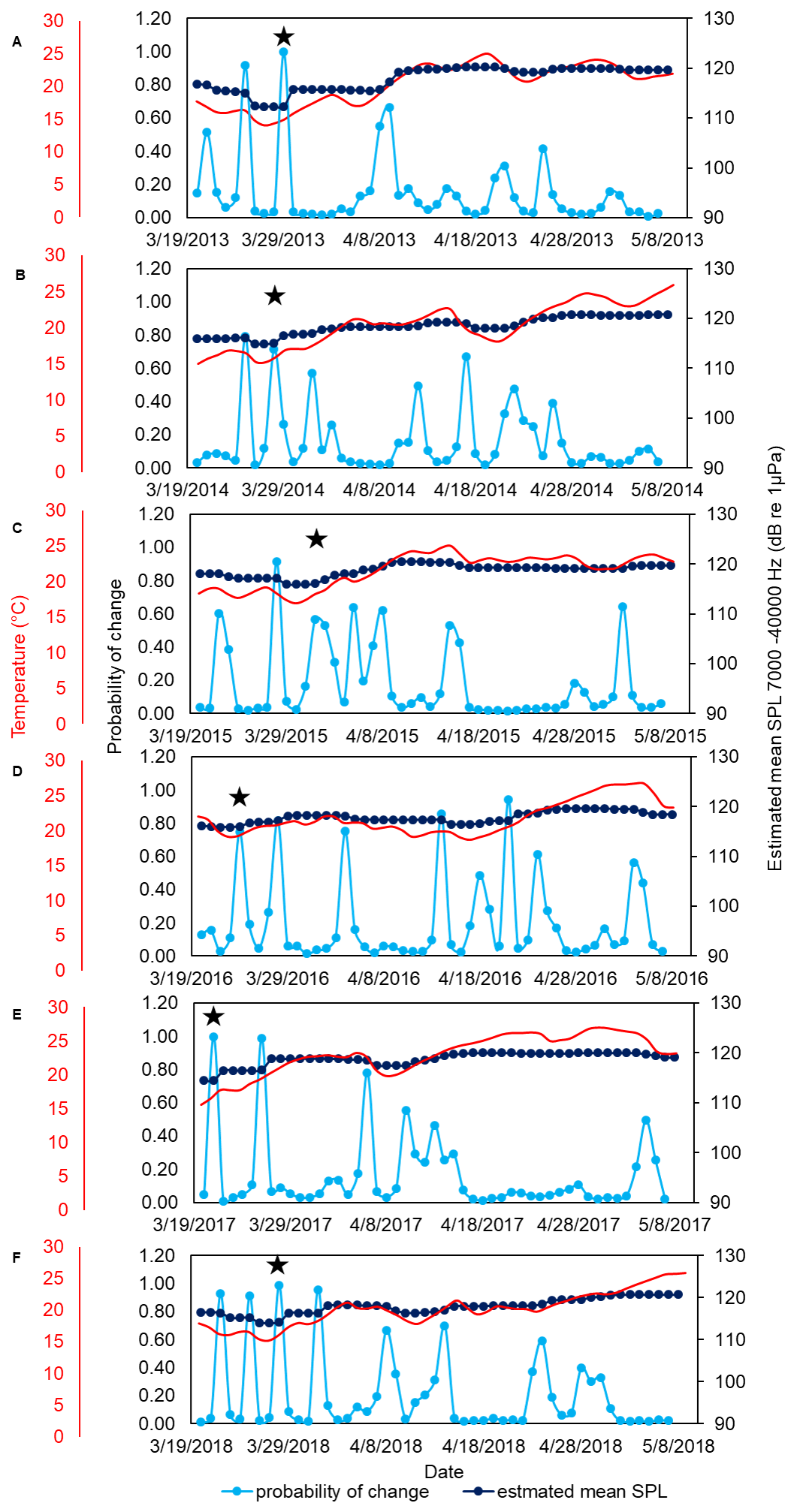

Supplement: S8 Fig — Probability of change during springtime (light blue line) with corresponding estimated mean high (7000–40,000 Hz) frequency SPL (dark blue line) and corresponding water temperature (red line) in years (A) 2013, (B) 2014, (C) 2015, (D) 2016, (E) 2017, and (F) 2018. Stars indicate first positive (i.e. detected change in estimated mean SPL due to an increase not a decrease in SPL values) PP ⩾ 0.5. (TIF) [file pone.0236874.s008.tif]

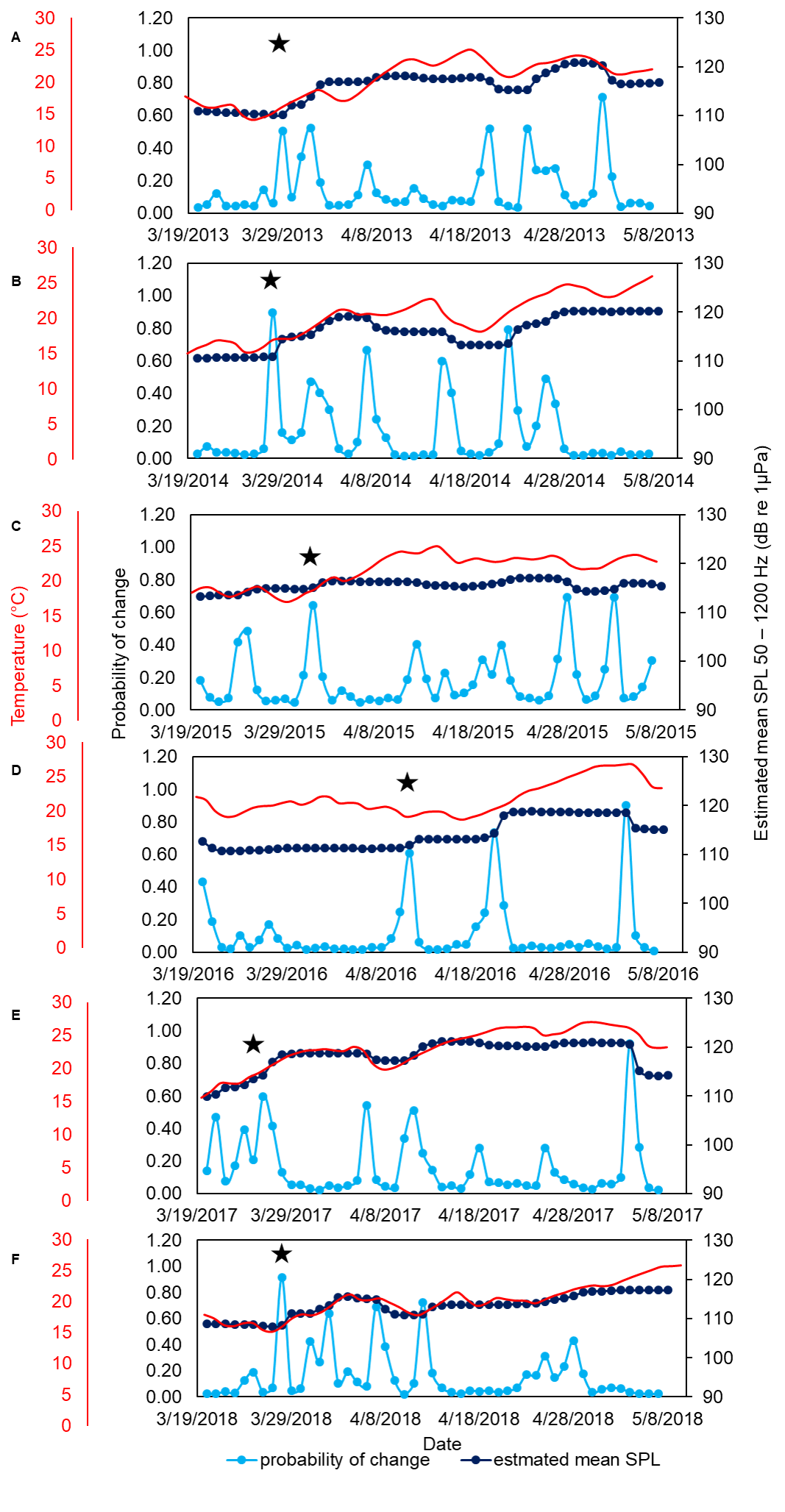

Supplement: S9 Fig — Probability of change during springtime (light blue line) with corresponding estimated mean low (50–1200 Hz) frequency SPL (dark blue line) and corresponding water temperature (red line) in years (A) 2013, (B) 2014, (C) 2015, (D) 2016, (E) 2017, and (F) 2018. Stars indicate first positive (i.e. detected change in estimated mean SPL due to an increase not a decrease in SPL values) PP ⩾ 0.5. (TIF) [file pone.0236874.s009.tif]

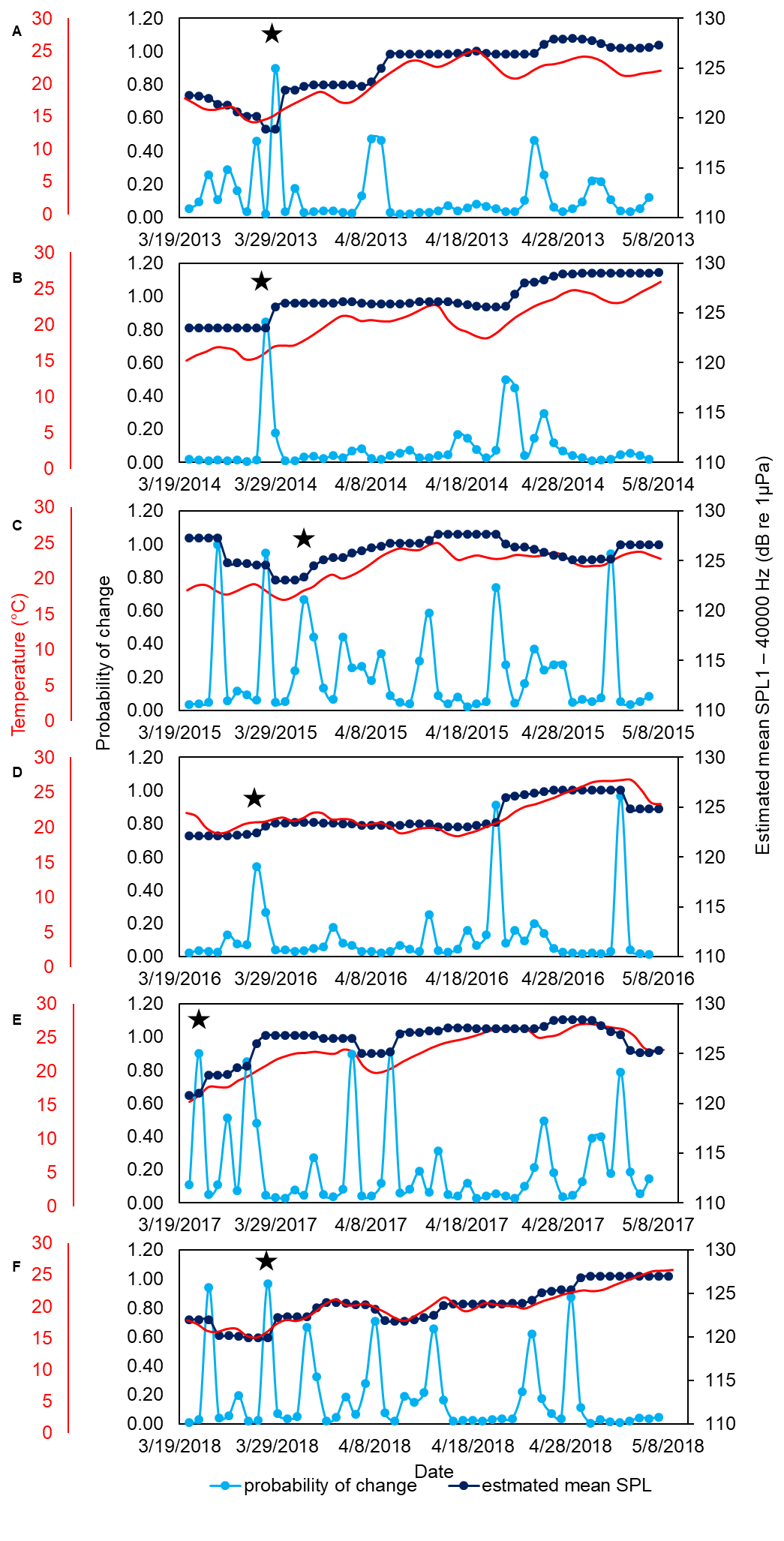

Supplement: S10 Fig — Probability of change during springtime (light blue line) with corresponding estimated mean (1–40,000 Hz) broadband frequency SPL (dark blue line) and corresponding water temperature (red line) in years (A) 2013, (B) 2014, (C) 2015, (D) 2016, (E) 2017, and (F) 2018. Stars indicate first positive (i.e. detected change in estimated mean SPL due to an increase not a decrease in SPL values) PP ⩾ 0.5. (TIF) [file pone.0236874.s010.tif]

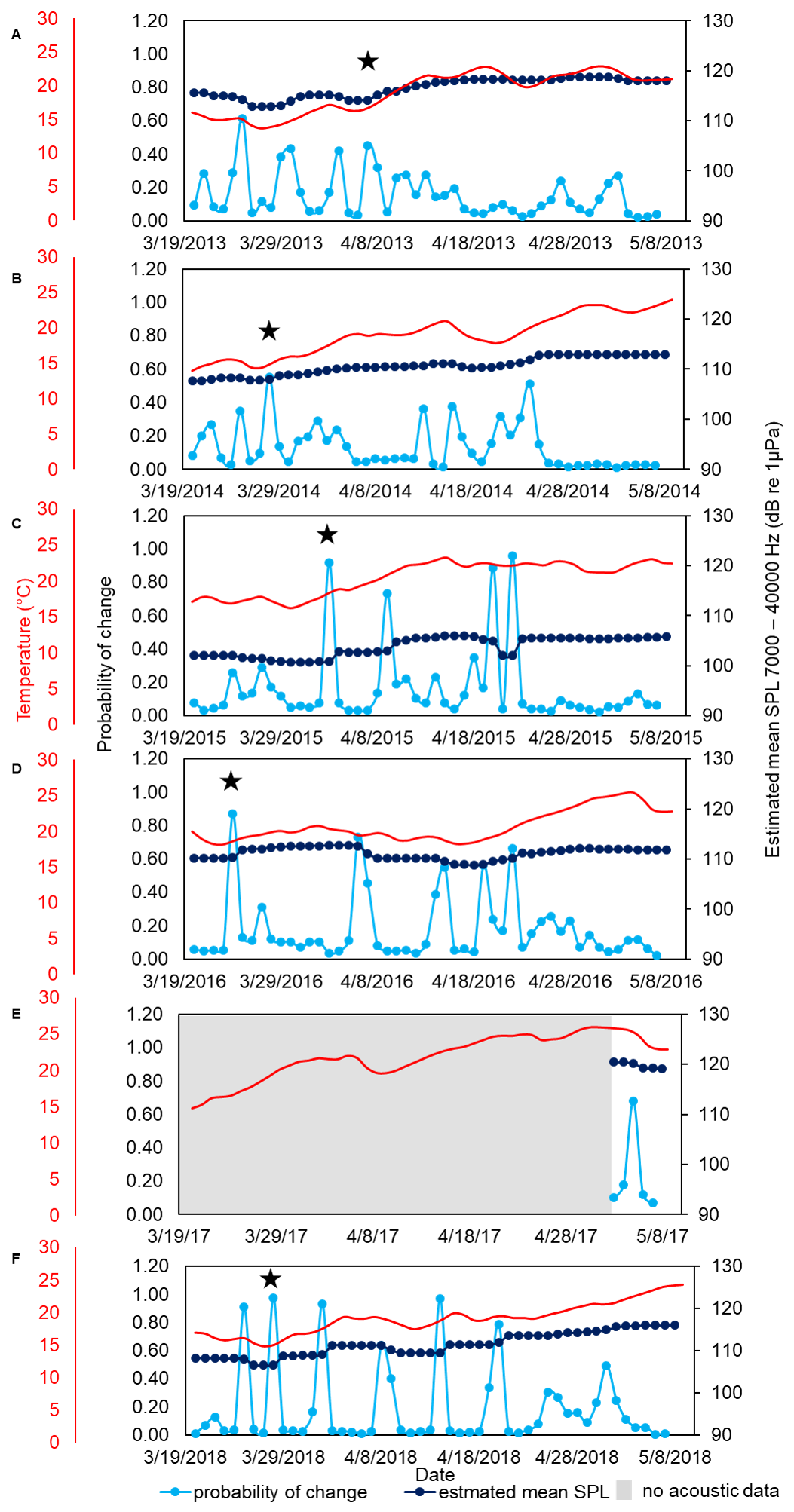

Supplement: S11 Fig — Probability of change during springtime (light blue line) with corresponding estimated mean high (7000–40,000 Hz) frequency SPL (dark blue line) and corresponding water temperature (red line) in years (A) 2013, (B) 2014, (C) 2015, (D) 2016, (E) 2017, and (F) 2018. Stars indicate first positive (i.e. detected change in estimated mean SPL due to an increase not a decrease in SPL values) PP ⩾ 0.5. Gray box = no data. (TIF) [file pone.0236874.s011.tif]

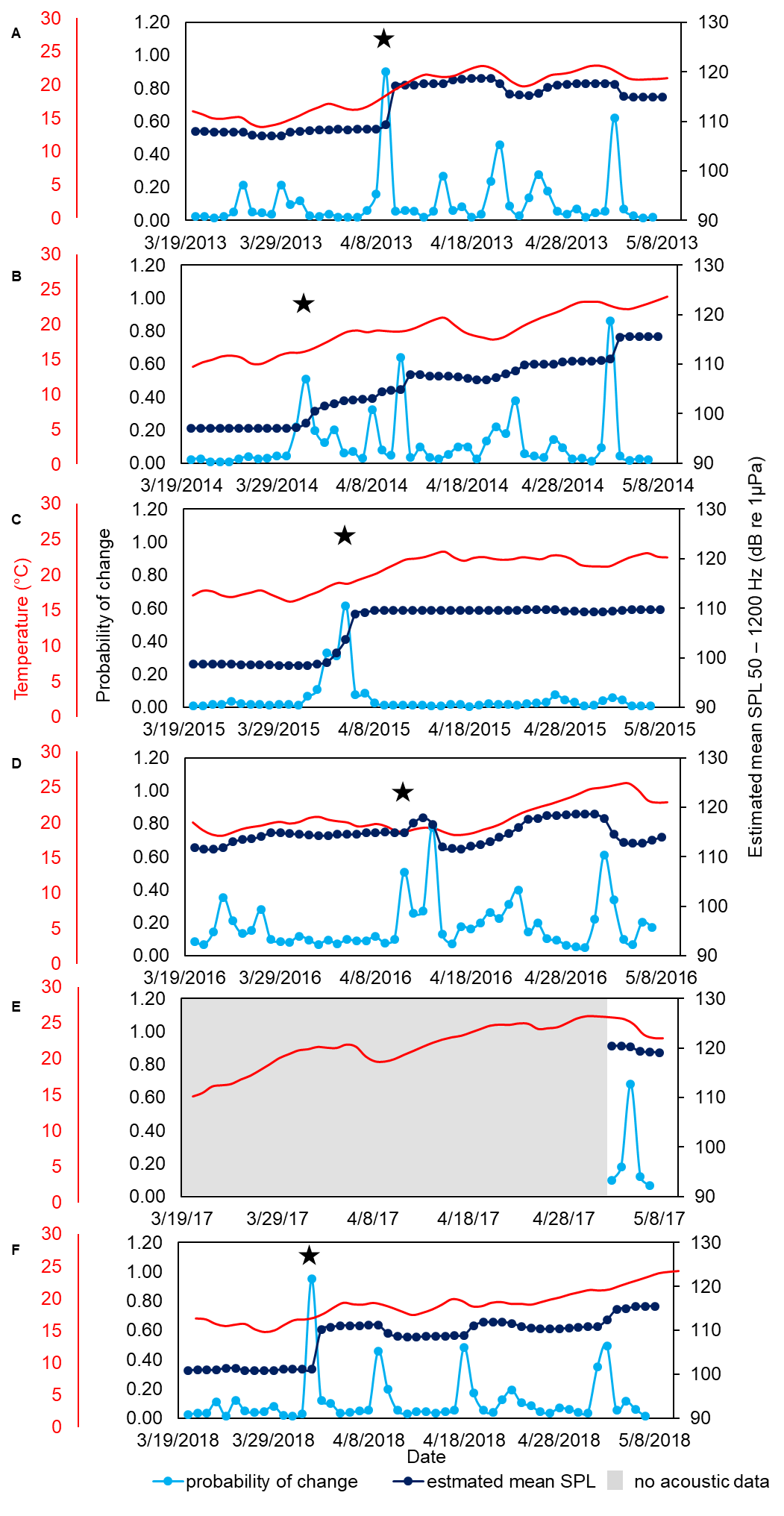

Supplement: S12 Fig — Probability of change during springtime (light blue line) with corresponding estimated mean low (50–1200 Hz) frequency SPL (dark blue line) and corresponding water temperature (red line) in years (A) 2013, (B) 2014, (C) 2015, (D) 2016, (E) 2017, and (F) 2018. Stars indicate first positive (i.e. detected change in estimated mean SPL due to an increase not a decrease in SPL values) PP ⩾ 0.5. Gray box = no data. (TIF) [file pone.0236874.s012.tif]

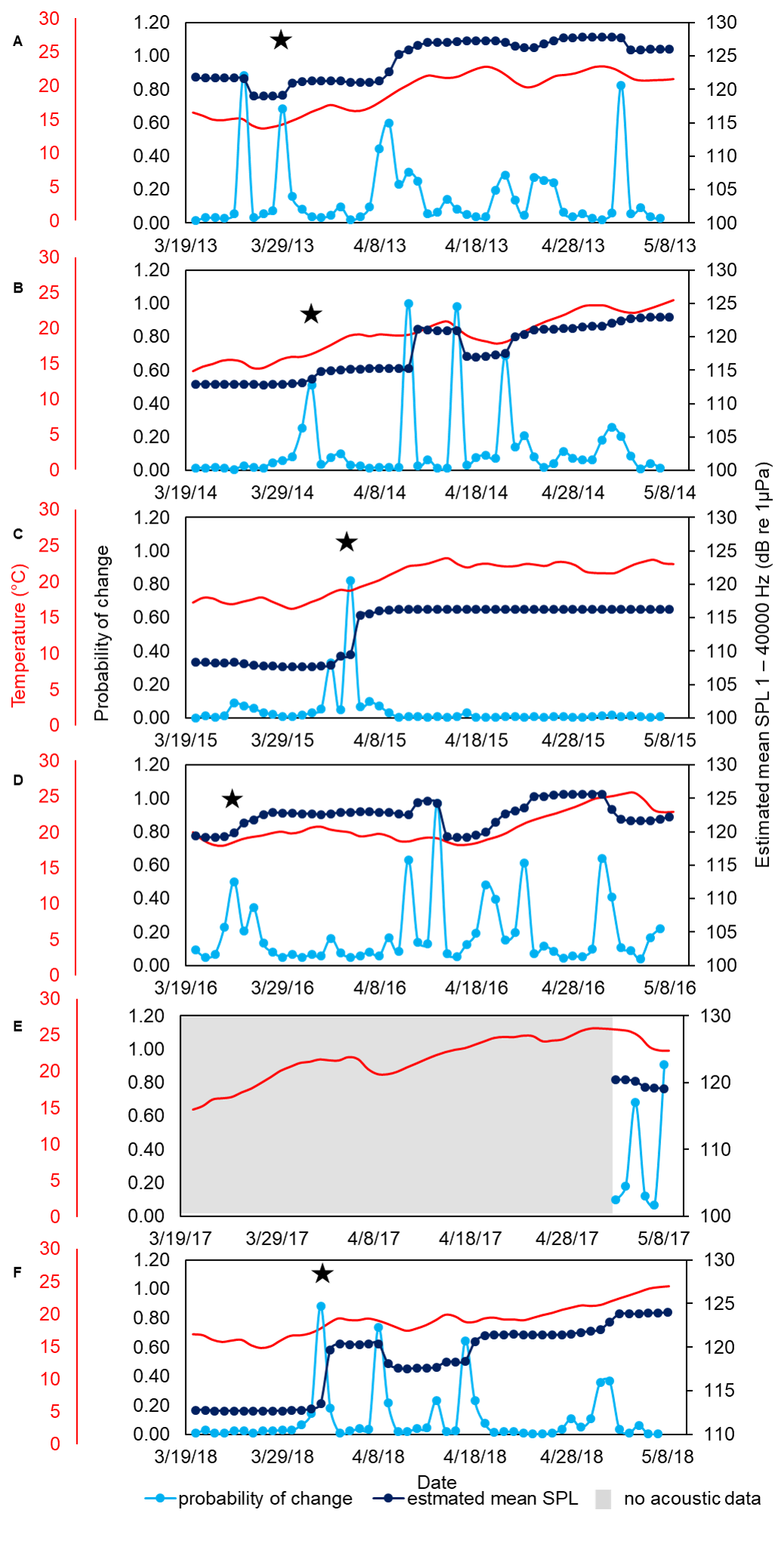

Supplement: S13 Fig — Probability of change during springtime (light blue line) with corresponding estimated mean broadband (1–40,000 Hz) frequency SPL (dark blue line) and corresponding water temperature (red line) in years (A) 2013, (B) 2014, (C) 2015, (D) 2016, (E) 2017, and (F) 2018. Stars indicate first positive (i.e. detected change in estimated mean SPL due to an increase not a decrease in SPL values) PP ⩾ 0.5. Gray box = no data. (TIF) [file pone.0236874.s013.tif]

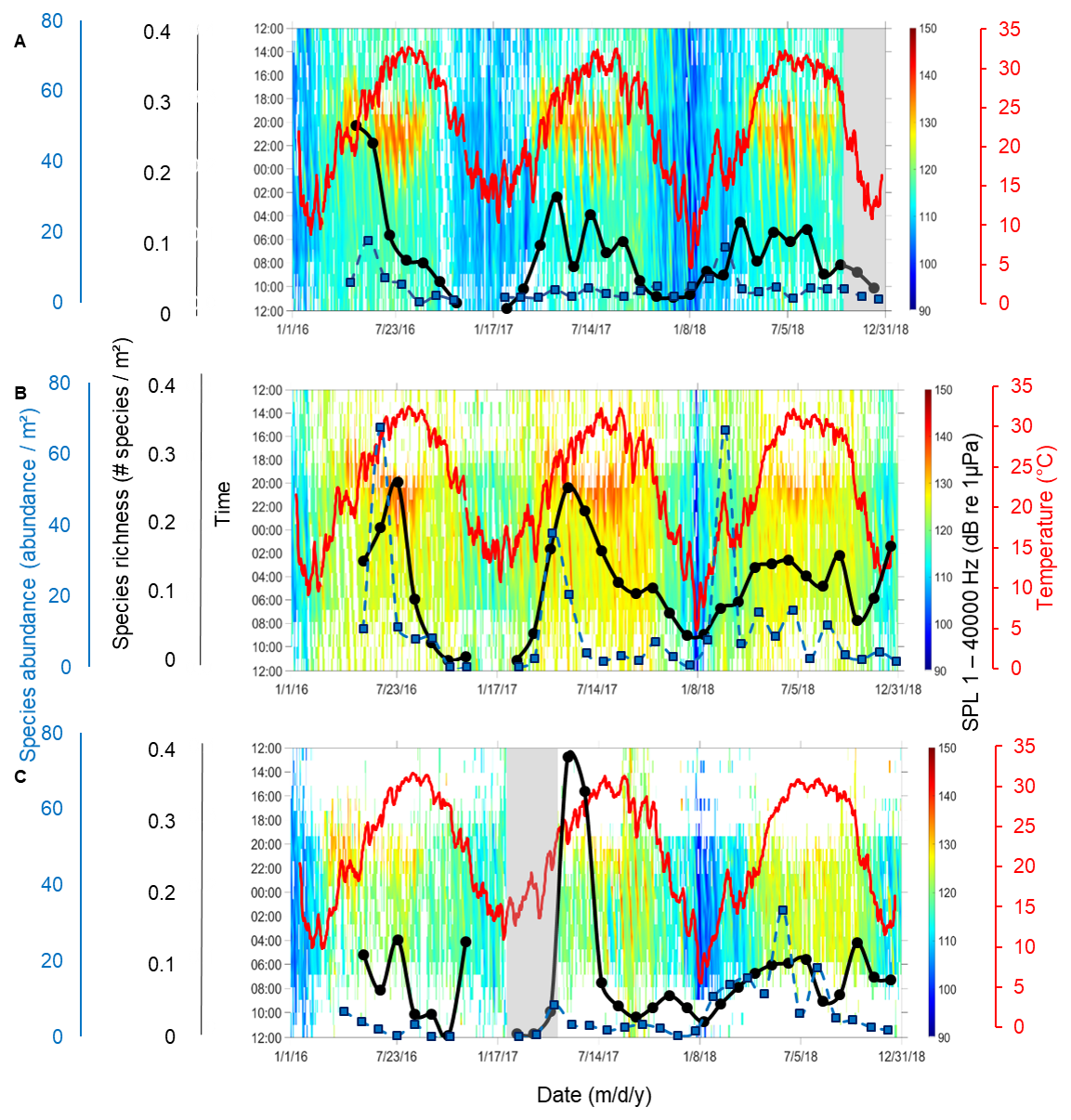

Supplement: S14 Fig — Heat maps represent temporal and spatial patterns of broadband (1–40,000 Hz) frequency SPLs with corresponding species richness (black line), species abundance (blue dotted line), and temperature (red line) at stations (A) 9M, (B) 14M, and (C) 37M. Files with physical sounds and anthropogenic noise removed = white and no data = gray box. (TIF) [file pone.0236874.s014.tif]

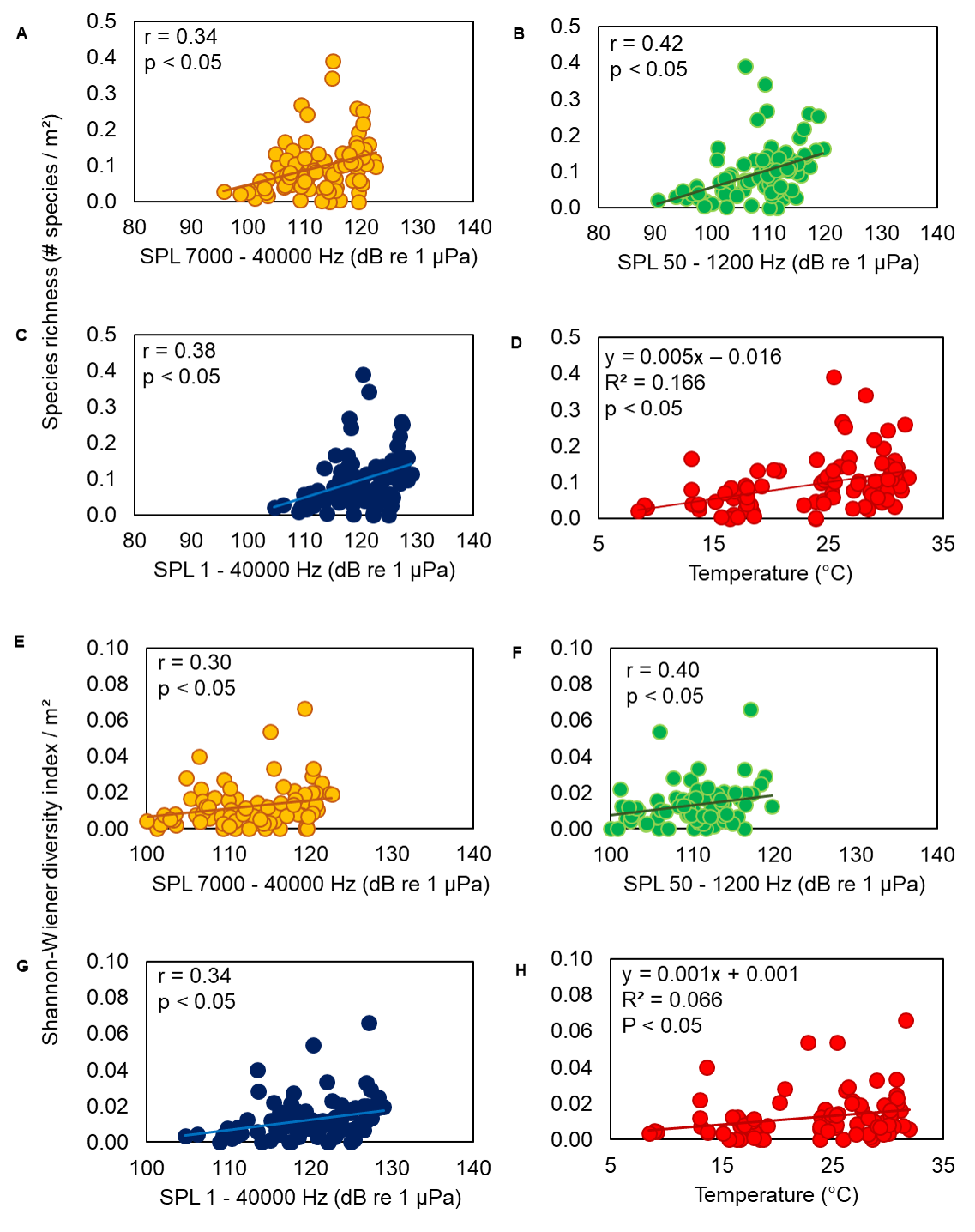

Supplement: S15 Fig — Pearson’s correlation between monthly averages of species richness and monthly averages of sound pressure levels (SPLs) in (A) high (7000–40,000 Hz), (B) low (50–1200 Hz), and (C) broadband (1–40,000 Hz) frequency ranges. Pearson’s correlation (r) between monthly averages of Shannon-Wiener diversity index and monthly averages SPLs in (E) high (7000–40,000 Hz), (F) low (50–1200 Hz), and (G) broadband (1–40,000 Hz) frequency ranges. Linear regression between monthly averages of temperature as the independent variable and monthly averages of (D) species richness and (H) Shannon-Wiener diversity index as dependent variables between 2016 and 2018 at all stations combined. For correlations N = 86 and for regression N = 90. (TIF) [file pone.0236874.s015.tif]

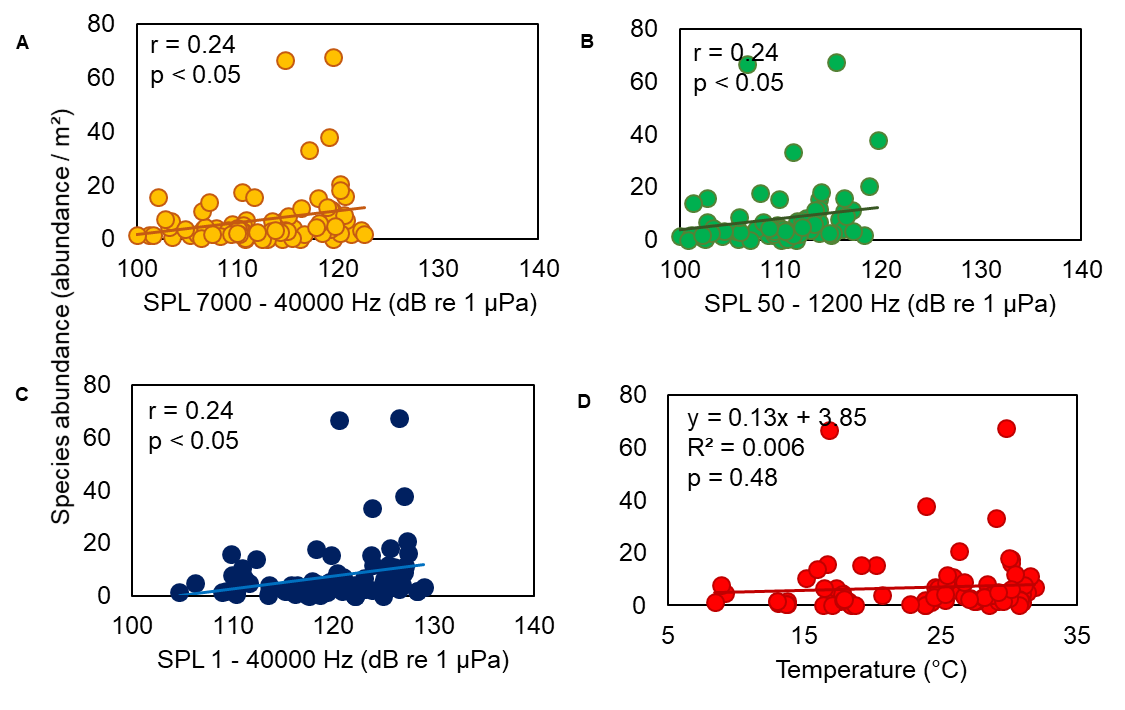

Supplement: S16 Fig — Pearson’s correlation between monthly averages of species richness and monthly averages of sound pressure levels (SPLs) in (A) high (7000–40,000 Hz), (B) low (50–1200 Hz), and (C) broadband (1–40,000 Hz) frequency ranges. Linear regression between monthly averages of temperature as the independent variable and monthly averages of (D) species abundance between 2016 and 2018 at all stations combined. For correlations N = 86 and for regression N = 90. (TIF) [file pone.0236874.s016.tif]
