# Supplementary material for: What’s all that racket! Soundscapes, phenology, and biodiversity in estuaries
Source: PLoS One. 2020 Sep 3;15(9):e0236874. doi: 10.1371/journal.pone.0236874 (PMC7470342; doi:10.1371/journal.pone.0236874)
Supplement: Attachment — Submitted filename: PONE-D-20-03292_SM.pdf [file pone.0236874.s025.pdf]

PLOS ONE

What's all that racket! Soundscapes, phenology, and biodiversity in estuaries

--Manuscript Draft--

**Manuscript Number:**	PONE-D-20-03292
**Article Type:**	Research Article
**Full Title:**	What's all that racket! Soundscapes, phenology, and biodiversity in estuaries
**Short Title:**	Estuarine soundscapes and biodiversity
**Corresponding Author:**	Eric W. Montie, Ph.D.<br>University of South Carolina Beaufort<br>Bluffton, SC UNITED STATES
**Keywords:**	Soundscape; phenology; climate; bioacoustics; estuary; fish sounds; snapping shrimp; diversity; species richness; passive acoustics
**Abstract:**	There is now clear evidence that climate change affects terrestrial and marine ecosystems and can cause phenological shifts in behavior. Utilizing sound to demonstrate phenology is gaining popularity in terrestrial environments. In marine ecosystems, this technique is yet to be used due to a lack of multiyear datasets. Our study demonstrate soundscape phenology in an estuary using a six-year dataset. In this study, we showed that an increase in acoustic activity of snapping shrimp and certain fish species occurred earlier in years with warmer springs. In addition, we combined passive acoustics and traditional sampling methods (seines) and detected positive relationships between temporal patterns of the soundscape and biodiversity. This study shows that passive acoustics can provide information on the ecological response of estuaries to climate variability.
**Order of Authors:**	Agnieszka Monczak
	Bradshaw McKinney
	Claire Mueller
	Eric W. Montie, Ph.D.
**Additional Information:**	
**Question**	**Response**
**Financial Disclosure**<br><br>Enter a financial disclosure statement that describes the sources of funding for the work included in this submission. Review the submission guidelines for detailed requirements. View published research articles from *PLOS ONE* for specific examples.<br><br>This statement is required for submission and **will appear in the published article** if the submission is accepted. Please make sure it is accurate.	This work was supported by Spring Island Trust, Town of Bluffton/Beaufort County, multiple University of South Carolina (USC) ASPIRE internal awards, Community Foundation of the Lowcountry, SC EPSCoR/IDeA Program award (#17-RE02), South Carolina Aquarium, Research Initiative for Summer Engagement (RISE) grant from USC, The LowCountry Institute, USCB Sea Islands Institute, Palmetto Bluff Conservancy, and the Port Royal Sound Foundation. This work was also supported, in part, by the Southeast Coastal Ocean Observing Regional Association (SECOORA) with NOAA financial assistance award number NA16NOS0120028. The statements, findings, conclusions, and recommendations are those of the author(s) and do not necessarily reflect the views of SECOORA or NOAA. All funds were awarded to EWM. The funders had no role in study design, data collection and analysis, decision to publish, or preparation of the manuscript.

Powered by Editorial Manager® and ProduXion Manager® from Aries Systems Corporation

**Unfunded studies**<br>Enter: *The author(s) received no specific funding for this work.*<br><br>**Funded studies**<br>Enter a statement with the following details:<br>• Initials of the authors who received each award<br>• Grant numbers awarded to each author<br>• The full name of each funder<br>• URL of each funder website<br>• Did the sponsors or funders play any role in the study design, data collection and analysis, decision to publish, or preparation of the manuscript?<br>• **NO** - Include this sentence at the end of your statement: *The funders had no role in study design, data collection and analysis, decision to publish, or preparation of the manuscript.*<br>• **YES** - Specify the role(s) played.<br><br>* typeset	
**Competing Interests**<br><br>Use the instructions below to enter a competing interest statement for this submission. On behalf of all authors, disclose any competing interests that could be perceived to bias this work—acknowledging all financial support and any other relevant financial or non-financial competing interests.<br><br>This statement **will appear in the published article** if the submission is accepted. Please make sure it is accurate. View published research articles from *PLOS ONE* for specific examples.	The authors have declared that no competing interests exist.

**NO authors have competing interests**<br><br>Enter: *The authors have declared that no competing interests exist.*<br><br>**Authors with competing interests**<br><br>Enter competing interest details beginning with this statement:<br><br>*I have read the journal's policy and the authors of this manuscript have the following competing interests: [insert competing interests here]*<br><br>* typeset	
**Ethics Statement**<br><br>Enter an ethics statement for this submission. This statement is required if the study involved:<br><br>• Human participants<br>• Human specimens or tissue<br>• Vertebrate animals or cephalopods<br>• Vertebrate embryos or tissues<br>• Field research<br><br>Write "N/A" if the submission does not require an ethics statement.<br><br>General guidance is provided below. Consult the submission guidelines for detailed instructions. **Make sure that all information entered here is included in the Methods section of the manuscript.**	This work was conducted under South Carolina Department of Natural Resources permit numbers 5135 and 5136 and USC IACUC protocol 2233-101181-022217.

**Format for specific study types**<br><br>**Human Subject Research (involving human participants and/or tissue)**<br>• Give the name of the institutional review board or ethics committee that approved the study<br>• Include the approval number and/or a statement indicating approval of this research<br>• Indicate the form of consent obtained (written/oral) or the reason that consent was not obtained (e.g. the data were analyzed anonymously)<br><br>**Animal Research (involving vertebrate animals, embryos or tissues)**<br>• Provide the name of the Institutional Animal Care and Use Committee (IACUC) or other relevant ethics board that reviewed the study protocol, and indicate whether they approved this research or granted a formal waiver of ethical approval<br>• Include an approval number if one was obtained<br>• If the study involved *non-human primates*, add *additional details* about animal welfare and steps taken to ameliorate suffering<br>• If anesthesia, euthanasia, or any kind of animal sacrifice is part of the study, include briefly which substances and/or methods were applied<br><br>**Field Research**<br><br>Include the following details if this study involves the collection of plant, animal, or other materials from a natural setting:<br>• Field permit number<br>• Name of the institution or relevant body that granted permission	
**Data Availability**<br><br>Authors are required to make all data underlying the findings described fully available, without restriction, and from the time of publication. PLOS allows rare exceptions to address legal and ethical concerns. See the PLOS Data Policy and FAQ for detailed information.	Yes - all data are fully available without restriction

A Data Availability Statement describing where the data can be found is required at submission. Your answers to this question constitute the Data Availability Statement and **will be published in the article**, if accepted.<br><br>**Important:** Stating 'data available on request from the author' is not sufficient. If your data are only available upon request, select 'No' for the first question and explain your exceptional situation in the text box.<br><br>Do the authors confirm that all data underlying the findings described in their manuscript are fully available without restriction?	
**Describe where the data may be found in full sentences. If you are copying our sample text, replace any instances of XXX with the appropriate details.**<br><br>• If the data are **held or will be held in a public repository**, include URLs, accession numbers or DOIs. If this information will only be available after acceptance, indicate this by ticking the box below. For example: *All XXX files are available from the XXX database (accession number(s) XXX, XXX.).*<br>• If the data are all contained **within the manuscript and/or Supporting Information files**, enter the following: *All relevant data are within the manuscript and its Supporting Information files.*<br>• If neither of these applies but you are able to provide **details of access elsewhere**, with or without limitations, please do so. For example:<br><br>*Data cannot be shared publicly because of [XXX]. Data are available from the XXX Institutional Data Access / Ethics Committee (contact via XXX) for researchers who meet the criteria for access to confidential data.*<br><br>*The data underlying the results presented in the study are available from (include the name of the third party*	All the original data, materials, and codes are preserved and retrievable for reanalysis. In total, we collected 392,106 raw wav files from all three stations that are stored on network drive and backed up on ioSafe external drives (ioSafe HQ Roseville, CA, USA). These sound files are available on request. In addition, we have stored the raw sound pressure levels and seine fishing data in Excel datasheets as well as the original Matlab and R codes used during analysis that are available. We indicated any missing data on each figure and figure legend. We did not remove any data from formal analysis.

Powered by Editorial Manager® and ProduXion Manager® from Aries Systems Corporation

and contact information or URL).<br>• This text is appropriate if the data are owned by a third party and authors do not have permission to share the data.<br><br>* typeset	
Additional data availability information:	Tick here if your circumstances are not covered by the questions above and you need the journal's help to make your data available.

Manuscript
Click here to download Manuscript Monczak et al_Soundscape and biodiversity_020420.docx

What's all that racket! Soundscapes, phenology, and biodiversity in estuaries

Agnieszka Monczak[a,b,¶], Bradshaw McKinney[a], Claire Mueller[a], and Eric W. Montie[a, ¶,*]

[a] Department of Natural Sciences, University of South Carolina Beaufort, Bluffton, South Carolina, USA

[b] Institute of Biological and Environmental Sciences, University of Aberdeen, Aberdeen, UK

*Corresponding author

E-mail: emontie@uscb.edu (EWM)

[¶]These authors contributed equally to this manuscript

Abstract

There is now clear evidence that climate change affects terrestrial and marine ecosystems and can cause phenological shifts in behavior. Utilizing sound to demonstrate phenology is gaining popularity in terrestrial environments. In marine ecosystems, this technique is yet to be used due to a lack of multiyear datasets. Our study demonstrate soundscape phenology in an estuary using a six-year dataset. In this study, we showed that an increase in acoustic activity of snapping shrimp and certain fish species occurred earlier in years with warmer springs. In addition, we combined passive acoustics and traditional sampling methods (seines) and detected positive relationships between temporal patterns of the soundscape and biodiversity. This study shows that passive acoustics can provide information on the ecological response of estuaries to climate variability.

Introduction

Over the past decades, researchers have reported changes in the cyclic nature of migratory and breeding patterns in fauna (e.g. insects, amphibians, and birds) associated with climate change [1-3]. It is important to monitor these shifts because the response of individual species may vary and could disrupt interactions with other species, leading to ecosystem imbalance (e.g. trophic mismatch) [4, 5]. Current advances in passive acoustic monitoring allow for long-term assessments of soundscapes, which can provide instrumental information on biological processes [6]. Long-term monitoring of sound can provide information on the timing of recurring phenomena (e.g. migration, foraging, and spawning) and can detect shifts in these

biological processes [7, 8]. In marine ecosystems, there seems to be a knowledge-gap in understanding soundscape phenology, simply because there are few long-term datasets. Soundscape ecology is evolving with novel technological advances, providing alternative approaches in assessing behavior as well as community structure, function, and dynamics [6]. Recently, there has been an increasing interest in assessing whether passive acoustics can capture terrestrial biodiversity [e.g. 9, 10]. Applications to marine ecosystems are more challenging but evidence suggests that sound diversity can reflect species diversity (i.e. measured by underwater visual census of fish) in mangrove, coral reef, seagrass, and rocky habitats [11, 12]. In the Southeast USA, significant contributors of biological sound to estuarine ecosystems include snapping shrimp (genus Alpheus and Synalpheus) and fish (families Batrachoididae and Sciaenidae). Calls are associated mainly with defense and foraging (e.g. snapping shrimp) as well as courtship (e.g. fish) [e.g. 13-17]. Many sound-producing species use estuaries periodically for spawning and nurseries, and sound / species richness can vary seasonally [18, 19].

In this study, we deployed three passive acoustic recorders for six years (February 2013 - December 2018) in the May River, SC, US. In addition, we used a catch and release method (i.e. haul seines) to assess species diversity and abundance in intertidal creeks located in close proximity to recording platforms. The specific objectives were to: (i) determine temporal patterns of high, low, and broadband frequency sound pressure levels (SPLs) over the six year time span; (ii) determine how certain environmental factors influence SPLs; (iii) examine phenology of acoustic activity of snapping shrimp and sound producing fish species (i.e. measured as changes in high and low SPLs, respectively); and (iv) determine temporal patterns of species diversity and abundance and examine how these indices correlate with the soundscape.

Results

Temporal patterns of the estuarine soundscape

Comparisons of high (7000 – 40,000 Hz), low (50 – 1200 Hz), and broadband (1 – 40,000 Hz) frequency SPLs from 2013 to 2018 revealed temporal and spatial differences (Figs. 1 and 2, S1 Fig.) [20]. High frequency SPLs reflected the acoustic activity of snapping shrimp; low frequency SPLs reflected acoustic activity of soniferous fish species and the lower portion of snapping shrimp snaps; while broadband included all the sounds of biological origin. We observed distinct temporal patterns in SPLs that were influenced by location, year, lunar phase, day/night, tidal phase, temperature, day length, and rainfall. From the three random forest models tested, the models including temperature explained the most variance in the data as compared to models that included rainfall or day length as a factor (S1 and S2 Tables) [20]. The designed models with temperature explained 88%, 54%, and 68% of the data variability for high, low, and broadband SPL, respectively (S1 Table). We applied the same models to the data that excluded anthropogenic noise, and we observed similar results (i.e. models that included temperature as a factor explained 90%, 56%, and 71% of the data variability for high, low, and broadband SPL, respectively) (Table S2) [20]. The factors that were most significant in influencing SPL were location, temperature, and year (S1 and S2 Tables). We observed significant differences in SPL values of biological origin among stations with 14M having the highest values and 9M the lowest ($p < 0.05$) [20]. In addition, we detected the highest contribution of anthropogenic noise at station 37M (i.e. near the Intracoastal Waterway; 11% of files analyzed) and the lowest at station 9M (i.e. near the headwaters; 2% of files analyzed) (S2-S4 Figs.). The anthropogenic noise detected was the most prevalent during the day in the summer months (S2-S4 Figs.). One striking pattern that we observed in SPL fluctuations was how SPL values increased and

decreased with the seasonal temperature changes of the estuary (Figs. 1, 2, and S1 Fig.). With an increase of water temperature for every 0.5°C, 0.6°C, and 0.8°C during the springtime, we detected a corresponding increase of 1 dB in SPL for high, low, and broadband frequencies, respectively. Higher values of SPLs were present in the summer months as compared to lower values in the winter, early spring, and late fall. We observed significant differences in SPLs among years for all examined frequency ranges ($p < 0.05$). The highest SPL values occurred in 2013 and 2014, the lowest in 2016 and 2017. In addition, snapping shrimp acoustic activity was higher during the day, low tide, and new moon as compared to the night, high tide, and full moon ($p < 0.05$). SPLs within the low frequency band were higher during the night (i.e. associated with fish chorusing) and followed an oscillating pattern associated with the lunar phase with higher values recorded on the first quarter of the lunar phase ($p < 0.05$). Values in the broadband SPL frequency range, which reflected a combination of snapping shrimp and fish acoustic activity, were the highest during the night, new moon and falling tide ($p < 0.05$).

Fig. 1. Time series of high frequency sound pressure levels (SPL) from 2013 to 2018. Heat maps represent temporal and spatial patterns of high (i.e. 7000 – 40,000 Hz) frequency SPLs reflecting snapping shrimp acoustic activity at stations (A) 9M, (B) 14M, and (C) 37M in the May River, SC. Time is shown between noon and noon of the next day. Gaps in data = white, temperature = black line, and daylight hours = dotted line. Green dots indicate first posterior probability (PP) of change $\geqslant 0.5$ detected during springtime. At station (C) 37M first PP was not calculated for spring 2017 due to missing acoustic data.

Fig. 2. Time series of low frequency sound pressure levels (SPLs) from 2013 until 2018. Heat maps represent temporal and spatial patterns of low (i.e. 50 – 1200 Hz) frequency SPLs reflecting fish and lower frequency range of snapping shrimp acoustic activity at stations (A) 9M, (B) 14M, and (C) 37M in the May River, SC. Time is shown between noon and noon of the next day. Gaps in data = white, temperature = black line, and daylight hours = dotted line. Green dots indicate first posterior probability (PP) of change ⩾ 0.5 detected during springtime. At station (C) 37M first PP was not calculated for spring 2017 due to missing acoustic data.

Soundscape phenology

Based on six years of data recorded at three stations, we examined the phenology of acoustic activity of snapping shrimp (as a measure of high frequency SPL) and fish (as a measure of low frequency SPL) by detecting the date of the first abrupt change in SPL (i.e. posterior probability or PP ≥ 0.5) [20]. In spring recordings at station 9M, we found the first peak in high frequency SPL to occur on April 9, April 3, April 1, March 25, March 23, and March 29 in 2013, 2014, 2015, 2016, 2017, and 2018, respectively (S3 Table S3 and S5 Fig.). At the same station, we detected the first peak in low frequency SPL to occur on April 2, March 27, April 6, March 25, March 25, and March 28 in 2013, 2014, 2015, 2016, 2017, and 2018, respectively (S4 Table and S6 Fig.). Similar patterns were found at stations 14M and 37M (S3 and S4 Tables, S8-S13 Figs.). We found that in years with higher mean spring water temperatures, the first peak in high, low, and broadband SPL occurred earlier as compared to years with lower mean spring water temperatures (Fig. 3 A-C). During the spring of 2017, an increase in acoustic activity of snapping shrimp was detected 8 days earlier than the 6-year average at station 9M (S3 Table). At the same station, during spring of 2017, acoustic activity of soniferous fish species was detected 4 days

earlier than the 6-year average (S5 Table). Mean water temperature during spring of 2017 was the highest (i.e. + 1.21°C than the 6 year average) of all 6 years monitored (S3 Table). On the other hand, the mean spring temperature in 2013 was the lowest (i.e. -1.33°C than the 6 year average), and the first peak in snapping shrimp acoustic activity was detected 9 days later than the 6 year average at station 9M, while fish acoustic activity was detected 4 days later. We found similar patterns at stations 14M and 37M (S3 and S4 Tables). We found negative correlations between mean spring water temperature and the timing of the first peak in probability of change for high, low, and broadband SPL at stations 9M and 14M (Fig. 3 D-F). In addition, during the winter of 2017-2018, we recorded the lowest, minimum water temperature and the lowest SPL values of all three winters monitored (Figs. 1-2). During the spring 2018, the first peak in SPLs was detected later than in the years with higher winter and spring temperatures (S3 and S4 Tables). In the years with higher fluctuations in spring water temperature, there were more abrupt changes in SPLs (S8-S13 Figs.).

Fig. 3. Relationship between mean spring water temperature and day of year of first posterior probability (PP) of change ⩾ 0.5.

Left panels: lines with dots represent day of year of first PP ⩾ 0.5 of sound pressure level in (A) high (7000 – 40,000 Hz), (B) low (50 – 1200 Hz), and (C) broadband (1 – 40,000 Hz) frequency range while lines with squares represent mean spring water temperature at stations 9M and 14M. Right panels: relationship between mean spring water temperature and day of year of first PP ⩾ 0.5 of sound pressure level in (D) high (7000 – 40,000 Hz), (E) low (50 – 1200 Hz), and (F) broadband (1 – 40,000 Hz) frequency range with corresponding mean spring water temperature at stations 9M and 14M.

Soundscape and biodiversity

We used haul seines to estimate species richness, the Shannon-Wiener diversity index, and total abundance of species in the May River estuary between 2016 and 2018 [20]. In total, we caught 5 species of invertebrates and 54 species of fish during seining (S5 Table). We detected temporal patterns in species richness, the Shannon-Wiener diversity index, and abundance for invertebrates and fish species with a peak during springtime and minimum during wintertime. This temporal pattern followed the warming and cooling patterns of the estuary as well as the oscillating pattern of the biological soundscape (Fig. 4 and S14 Fig.). We found significant positive correlations between species richness (as well as the Shannon-Wiener diversity index and abundance) with high, low, and broadband SPLs (S15 A-C, E-H and S16 A-C Figs.); the highest correlations occurred with low frequency SPL (S15 B and F Fig.) [20]. In addition, we found a significant positive regression between water temperature and species richness (as well as Shannon-Wiener diversity index) but not between temperature and species abundance (S15 D, H, and S16 D Figs.). Spatially, overall years, the highest species richness, Shannon-Wiener Diversity index, and species abundance occurred at station 14M, where we observed the highest SPL values.

Fig. 4. Time series of broadband frequency sound pressure levels (SPLs), species richness, and species abundance from 2016 until 2018.

Heat maps represent temporal and spatial patterns of broadband (1 – 40,000 Hz) frequency SPLs reflecting all biological sounds with corresponding species richness (black line), species

abundance (blue dash line), and temperature (red line) at stations (A) 9M, (B) 14M, and (C) 37M in the May River. Gaps in data = white.

Discussion

In this study, we used a six-year passive acoustic dataset to understand the annual and inter-annual variability of an estuarine soundscape. Our findings show a strong relationship between temporal changes in acoustic activities of estuarine organisms and environmental factors. We showed that the transition between winter and spring is a dynamic time-period with an increase in biological sound during the spring, which mirrors the increase in phytoplankton, zooplankton, invertebrates, and fish abundance that drive changes in primary, secondary, and tertiary productivity within estuaries [21, 22]. In years with warmer spring temperatures, this seasonal transition occurred earlier than in years with cooler spring temperatures. This means that temperature plays an important factor in initiating certain behaviors (e.g. spawning), and earlier occurrences of these behaviors reflect an organismal response to climate variability [8]. In our study, we showed that sound production in snapping shrimp and fish (in families Batrachoididae and Sciaenidae) could serve as potential indicators of climate driven changes in spring phenology. In fact, snapping shrimp respond very quickly to changes in temperature with increased snapping activity with warmer temperatures and decreased activity with cooler temperatures [23]. Warmer temperatures have also potential to impact spawning phenology of certain fish species that have temperature-dependent gonadal development [24]. Our studies have shown that positive temperature anomalies increase sound production, while negative temperature anomalies decrease calling of fish belonging to families Batrachoididae and Sciaenidae [25]. Similar to other studies, we detected a significant increase in snapping shrimp

and fish acoustic activity in the spring and summer and a significant decrease in the fall and winter [e.g. 21-23, 26, 27]. These temporal variations in biological sound levels indicate that there is a strong connection between sound production and the seasonal changes in estuarine diversity and productivity.

Louder habitats may correlate with higher species richness and abundance [11, 12]. In the present study, we showed that higher species diversity and abundance occurred during seasonal periods (i.e. spring and summer) when biological sound levels in the low and high frequency bandwidths were the highest. Furthermore, biologically louder areas of the tidal river had higher diversity and abundance of invertebrates and fish. It is possible that the soundscape could provide organisms with information about habitat quality, resources, and potential predators [6]. It is also possible that the myriad of snapping shrimp and fish vocalizations guide organisms (e.g. larva, fish, and marine mammals) into and within the estuary. Recent laboratory and field playback experiments conducted in St. Johns, US Virgin Islands and Pamlico Sound, North Carolina, US have reported that larva utilize sound cues to find coral / oyster reefs based on the biogenic sound production of organisms occupying these habitats [28 – 30]. Habitat-specific sound characteristics may reflect an important habitat selection cue in driving settlement and recruitment patterns in marine communities, leading to higher biodiversity and potentially healthier habitats [29].

While our study investigated temporal changes of the soundscape and diversity over a relatively short timeframe (~ six years), this approach provides a blueprint for implementation over longer time scales [3]. Listening to soundscapes has the potential to provide insight into the response and resiliency of individual species and their behaviors. Integrating long-term soundscape characterization into coastal marine observatory networks would be powerful

because of its utility in providing acoustic behavior measurements at multiple levels of biological complexity (i.e. from snapping shrimp to fish to marine mammals) at time scales that range from minutes to years. This approach allows us to eavesdrop on key behaviors that can change rapidly or gradually in response to environmental changes and human use; thus it has potential to provide a measure of resilience or shifting baselines in a globally changing environment.

Materials and Methods

Study area

The May River (32°12’49’’N, 80°52’23”W) is located inland of the southern South Carolina coast (Fig. S1). This large subtidal river is ~22 km long and ~0.01 km wide near the source, and ~1 km wide at the mouth. The river is bordered by extensive patches of smooth cordgrass *Spartina alterniflora* and oyster reefs (i.e. eastern oyster *Crassostrea virginica*) with the town of Bluffton on the north-eastern side and Hilton Head Island at the mouth of the river. Water depth ranges from ~3 to ~7 m near the source and from ~4 to ~18 m near the mouth depending upon large semidiurnal tides. This area experiences a humid subtropical climate with hot summers and mild winters.

Fig. 5. Map of the May River, SC, USA.

Locations of stations 9M, 14M, and 37M that were acoustically monitored from February 2013 to December 2018 (blue) and seining locations monitored from May 2016 to December 2018 (yellow). (Inset) Location of the May River (black) in reference to the USA coast.

Data collection and analysis

We deployed DSG-Ocean recorders (Loggerhead Instruments, Sarasota, FL, USA), water level and temperature loggers (HOBO 100-Foot Depth Water Level Data Logger U20-001-02-Ti and HOBO Water Temperature Pro v2 U22-001, Onset Computer Corporation, Bourne, MA, USA) at stations 9M, 14M, and 37M between February 2013 and December 2018 following methods previously described (Fig. S1) [25]. Recorders collected sound samples for 2 min every 20 min at a sampling rate of 80 kHz over 22 deployments that were approximately 90 days long. For each 2 min wav file (i.e. a total of 392,106 files for all 3 stations), we determined the root mean square (rms) SPL for high (i.e. 7000 – 40,000 Hz), low (i.e. 50 – 1200 Hz), and broadband (i.e. 1 – 40,000 Hz) frequencies using custom scripts created in MATLAB R2017b (MathWorks, Inc., Natick, MA, USA). We created heat maps using MATLAB R2017b representing SPL values versus date and time at each acoustic station (i.e. 9M, 14M, and 37M) with corresponding water temperature and daylight hours. For manual analysis, we subsampled the data set (i.e. we analyzed files recorded on the hour; total of 130,702 files) and flagged all the files that contained anthropogenic noise. Then, we removed files that contained this type of sound (i.e. anthropogenic noise) and created heat maps representing only biological sounds with corresponding water temperature and daylight hours.

We performed invertebrate and fish sampling one to two times per month in the May River between 2016 and 2018 using a haul seine (i.e. seine width = 9.1 m, height = 1.2 m, and mesh diameter = 3 mm) and block nets (i.e. additional stationary seine nets to stop animals from escaping) at two to four locations per passive acoustic station (Fig. 5). This sampling equated to six to twelve seines per month selected randomly from a list of sites. Seine sites included tidal pools (i.e. shallow pools of water created on the low tide), intertidal creeks (i.e. small secondary

or tertiary creeks feeding from the main river accessible on the low tide), and sides of the river (i.e. stations located along the bank of the primary river). Before each seine, we recorded environmental parameters (i.e. water temperature, salinity, pH, and dissolved oxygen) using a YSI 556 Handheld Multiparameter Instrument (YSI Inc./Xylem Inc., Yellow Springs, OH, USA), and then we seined the area between the block nets. We measured the length and width of each seine for an area calculation. We transferred the catch into a live-well, quantified the abundance of each species, and then released live organisms at the original sampling location. We calculated average species richness per month based on the number of species in each seine standardized by the seine area. In addition, we calculated the average Shannon-Wiener diversity index divided by the area per month using package "vegan" in R version 3.4.2 (R Core Team, 2012) [31]. We calculated average species abundance per month based on the abundance of all the species in each seine standardized by the seine area. This work was conducted under South Carolina Department of Natural Resources permit numbers 5135 and 5136 and IACUC protocol 2233-101181-022217.

Statistical analyses

To assess the significance of specific factors in explaining variations in SPLs, we used package "Boruta", a wrapper algorithm based on the random forest algorithm in R [32 – 35]. Random forest models are non-parametric and do not require formal distribution assumptions. We used "permutation importance" rather than the default "mean decrease in impurity importance" to assess the importance of factors included in the model, since permutation importance is less biased of continuous and categorical variables with many levels [35 – 38]. In the final model, we set the specific parameters to: $p = 0.01$, $mtry = 2$, $ntree = 200$, $nodesize = 5$,

and set.seed = 42 [39, 40]. We included location, year, lunar phase, tide, day/night, temperature, day length, and rainfall as factors. We used four categories to differentiate the lunar and tidal cycle following methods previously described, and we used National Oceanic and Atmospheric Administration (NOAA) weather stations located close to the May River to obtain rainfall data for each day (S6 Table) [25]. Before applying the model, we tested the data for collinearity. Temperature, rainfall, and day length exhibited multi-collinearity that could bias the Boruta feature selection algorithm [41]. Hence, we created three different models for high, low, and broadband frequency SPL; in the first set of models, we included temperature as a factor; in the second set, we used day length; and in the third set, we used rainfall. Then, we compared the R^2 for each model that used a different factor, and we reported the models that best explained the variability of the data. We removed files that contained anthropogenic noise, and we followed the same approach to test variable importance. If categorical variables were significant, we applied Dunnett-Tukey-Kramer pairwise multiple comparison tests adjusted for unequal variances and unequal sample sizes with 95% confidence levels using package "DTK" in R to determine whether group means were significantly different from each other [42].

To identify significant changes in high, low, and broadband SPLs values during the springtime, we applied a Bayesian change point (BCP) analysis using package "bcp" in R [43, 44]. Bayesian change point analysis calculates the posterior probability (PP) for any given point in the time series that has an abrupt change. We defined a probability as significant when the change between the following and previous data point was $\geqslant$ 50% [8]. For each year, we reported the mean spring water temperature, date and day of year of the first significant PP, day anomaly (i.e. day of first significant PP for each year minus the six year average of days with the

first PP), and the value of the first significant PP at each station. We defined spring as the time between the astronomical vernal equinox and the summer solstice.

We tested for normality by investigating the distribution of the residuals, and center and dispersion of monthly averages of species richness, or Shannon-Wiener diversity index, and species abundance. We performed Pearson's correlations between monthly averages of species richness (or Shannon-Wiener diversity index and species abundance) and monthly averages of high, low, and broadband frequency SPLs for all stations combined. In addition, using linear regression, we tested the relationship between monthly averages of temperature as the independent variable and corresponding monthly averages of species richness (or Shannon-Wiener diversity index and species abundance) as the dependent variable. Correlations and regressions were performed in Matlab using two-sided hypothesis tests with a significance level of 0.05.

Data availability

All the original data, materials, and codes are preserved and retrievable for reanalysis. In total, we collected 392,106 raw wav files from all three stations that are stored on network drive and backed up on ioSafe external drives (ioSafe HQ Roseville, CA, USA). These sound files are available on request. In addition, we have stored the raw sound pressure levels and seine fishing data in Excel datasheets as well as the original Matlab and R codes used during analysis that are available. We indicated any missing data on each figure and figure legend. We did not remove any data from formal analysis.

Acknowledgments

We thank Bob and Lee Brewer of May River Plantation for their support and for allowing us to use their community dock for our University of South Carolina Beaufort (USCB) research vessel. Funding: This work was supported by Spring Island Trust, Town of Bluffton/Beaufort County, multiple University of South Carolina (USC) ASPIRE internal awards, Community Foundation of the Lowcountry, SC EPSCoR/IDeA Program award (#17-RE02), South Carolina Aquarium, Research Initiative for Summer Engagement (RISE) grant from USC, The LowCountry Institute, USCB Sea Islands Institute, Palmetto Bluff Conservancy, and the Port Royal Sound Foundation. This work was also supported, in part, by the Southeast Coastal Ocean Observing Regional Association (SECOORA) with NOAA financial assistance award number NA16NOS0120028. The statements, findings, conclusions, and recommendations are those of the author(s) and do not necessarily reflect the views of SECOORA or NOAA. Author contributions: Conceptualization: EWM, AM, BM; Data curation: AM; Formal Analysis: AM, CM, EWM; Funding acquisition: EWM; Investigation: EWM, AM, BM; Methodology: EWM, AM, BM; Project administration: EWM, AM; Resources: EWM; Supervision: EWM; Validation: EWM, AM; Visualization: AM, EWM; Writing – original draft: EWM, AM, CM; Writing – review & editing: EWM, AM, CM. Competing interests: Authors declare no competing interests.

References

1. J. L. Brown, S.H. Li, N. Bhagabati, Long-term trend toward earlier breeding in an American bird: a response to global warming? *Proc. Natl. Acad. Sci. U.S.A* 96, 5565–5569 (1999).

2. T. L. Root, J. T. Price, K. R. Hall, S. H. Schneider, Fingerprints of global warming on wild animals and plants (tier 2). *Nature* 421, 57–60 (2002).

3. G. Walther, E. Post, P. Convey, A. Menzel, C. Parmesan, T. J. Beebee, J. M. Fromentin, O. Hoegh-Guldberg, F. Bairlein, Ecological responses to recent climate change. *Nature* 416, 389−395 (2002).

4. M. Edwards, A. J. Richardson, Impact of climate change on marine phenology and tropic mismatch. *Nature* 430, 881-884 (2004).

5. S. K. Carter, D. Saenz, V. H. W. Rudolf, Shifts in phenological distributions reshape interaction potential in natural communities. *Ecol. Lett.* 21, 1143–1151 (2018).

6. B. C. Pijanowski, L. J. Villanueva-Rivera, S. L. Dumyahn, A. Farina A, B. L. Krause BL, B. M. Napoletano, S. H. Gage, N. Pieretti, Soundscape ecology: The science of sound in the landscape. *BioScience* 61, 203–216 (2011).

7. H. Lieth, (1974) “Purposes of a phenology book” in *Phenology and Seasonality Modeling* H Lieth, ed, (Springer-Verlag, New York, NY, 1974), pp. 3-19.

8. R. T. Buxton, E. Brown, L. Sharman, C. M. Gabriele, M.F. Mckenna, Using bioacoustics to examine shifts in songbird phenology. *Ecol. Evol.* 14, 1–14 (2016).

9. J. Sueur, S. Pavoine, O. Hamerlynck, S. Duvail, Rapid acoustic survey for biodiversity appraisal. *PloS One* 3, e4065 (2008).

10. M. W. Towsey, S. Parsons, J. Sueur, (2014) Ecology and acoustics at a large scale. *Ecol. Inform.* 21, 1-3 (2014).

11. E. Staaterman, M. B. Ogburn, A. H. Altieri, S. J. Brandl, R. Whippo, J. Seemann, M. Goodison, J .E. Duffy, Bioacoustic measurements complement visual biodiversity surveys: preliminary evidence from four shallow marine habitats. *Mar. Ecol. Prog. Ser.* 575, 207–215 (2017).

12. E. Desiderà, P. Guidetti, P. Panzalis, A. Navone, P. Boissery, C. Gervaise, L. D. Iorio, Acoustic fish communities: sound diversity of rocky habitats reflects fish species diversity. *Mar. Ecol. Prog. Ser.* 608, 183–197 (2019).

13. G. E. MacGinitie, Notes on the natural history of several marine Crustacea. *Am. Midi. Natural.* 18:1031–1036 (1937).

14. W. C. Guest, J.L. Lasswell, A note on courtship behavior and sound production of red drum. *Copeia* 1978, 337–338 (1978).

15. J. J. Luczkovich, R. C. Pullinger, S. E. Johnson, M. W. Sprague, Identifying sciaenid critical spawning habitats by the use of passive acoustics. *Trans. Am. Fish. Soc.* 137, 576–605 (2008).

16. E. W. Montie, C. Kehrer, J. Yost, K. Brenkert, T. O'Donnell, M. R. Denson, Long–term monitoring of captive red drum *Sciaenops ocellatus* reveals that calling incidence and structure correlate with egg deposition. *J. Fish. Biol.* 88, 1776–1795 (2016).

17. E. W. Montie, M. Hoover, C. Kehrer, J. Yost, K. Brenkert, T. O'Donnell, M. R. Denson, Acoustic monitoring indicates a correlation between calling and spawning in captive spotted seatrout (*Cynoscion nebulosus*). *PeerJ* 5:e2944, (2017) doi:10.7717/peerj.2944.

18. A. Monczak, C. Mueller, M. Miller, Y. Ji, S. Borgianini, E. W. Montie, Sound patterns of snapping shrimp, fish, and dolphins in an estuarine soundscape of the southeastern USA. *Mar. Ecol. Prog. Ser.* 609, 49-68 (2019).

19. L. A. Deegan, J. T. Finn, S. G. Ayvazian, C. A. Ryder-Kieffer, J. Buonaccorsi, Development and validation of an estuarine biotic integrity index. *Estuaries* 20, 601-617 (1997).

20. See materials and methods section.

21. L. W. Harding Jr, C. L. Gallegos, E. S. Perry, W. D. Miller, J. E. Adolf, M. E. Mallonee, H. Paerl, Long-term trends of nutrients and phytoplankton in Chesapeake Bay. *Estuaries and Coasts* 39, 664–681 (2015).

22. L. W. Harding Jr, M. E. Mallonee, E. S. Perry, W. D. Miller, J. E. Adolf, C. L. Gallegos, H. W. Paerl, Variable climatic conditions dominate recent phytoplankton dynamics in Chesapeake Bay. *Sci. Rep.* 6, 23773 (2016).

23. D. R. Bohnenstiehl, A. Lillis, D. B. Eggleston, The curious acoustic behavior of estuarine snapping shrimp: temporal patterns of snapping shrimp sound in sub–tidal oyster reef habitat. *PloS One* 11, 1–21 (2016).

24. K. McQueen, C. T. Marshall, Shifts in spawning phenology of cod linked to rising sea temperatures. *ICES J. Mar. Sci.* 74, 1561–1573 (2017).

25. A. Monczak, A. Berry, C. Kehrer, E. W. Montie, Long–term acoustic monitoring of fish calling provides baseline estimates of reproductive time–lines in the May River estuary, southeastern USA. *Mar. Ecol. Prog. Ser.* 581, 1–19 (2017).

26. J. J. Luczkovich, M. W. Sprague, S. E. Johnson, R. C. Pullinger, Delimiting spawning areas of weakfish, *Cynoscion regalis* (family Sciaenidae) in Pamlico Sound, North Carolina using passive hydroacoustic surveys. *Bioacoustics* 10, 143–160 (1999).

27. D. A. Mann, P. S. Lobel, Passive acoustic detection of sounds produced by the damselfish, *Dascyllus albisella* (Pomacentridae). *Bioacoustics* 6, 199–213 (1995).

28. M. J. A. Vermeij, K. L. Marhaver, C. M. Huijbers, I. Nagelkerken, D. Stephen D (2010) Coral larvae move toward reef sounds. PloS One 5, 3–6 (2010).

29. A. Lillis, D. B. Eggleston, D. R. Bohnenstiehl, Oyster larvae settle in response to habitat–associated underwater sounds. *PloS One* 8:e79337 (2013).

30. A. Lillis, A. Apprill, J. J. Suca, C. Becker, J. K. Llopiz, T. A. Mooney, Soundscapes influence the settlement of the common Caribbean coral *Porites astreoides* irrespective of light conditions. *R. Soc. Open. Sci.* 12(5), 181358 (2018).

31. J. Oksanen, F. G. Blanchet, M. Friendly, R. Kindt, P. Legendre, D. McGlinn, P. R. Minchin, R. B. O'Hara, G. L. Simpson, P. Solymos, M. H. H. Stevens, E. Szoecs, H. Wagner, vegan: Community Ecology Package. R package version 2.5-4 (2019).

32. M. B. Kursa, W. R. Rudnicki, Feature selection with the Boruta package. *J. Stat. Softw.* 36, 1-13 (2010).

33. M. B. Kursa, rFerns: an implementation of the random ferns method for general-purpose machine learning. *J. Stat. Softw.* 61, 10 (2014).

34. M. N. Wright, A. Ziegler, Ranger: a fast implementation of random forests for high dimensional data in C++ and R. *J. Stat. Softw*. 77, 1-17 (2017).

35. F. Degenhardt, S. Seifert, S. Szymczak, Evaluation of variable selection methods for random forests and omics data sets. *Brief Bioinform* 22, 492-503 (2019).

36. C. Strobl, A. L. Boulesteix, A. Zeileis, T. Hothorn, Bias in random forest variable importance measures: illustrations. Sources and a solution. *BMC Bioinformatics* 8, 25 (2007).

37. A. Altmann, L. Toloşi, O. Sander, T. Lengauer, Permutation importance: a corrected feature importance measure. *Bioinformatics* 26, 1340-7 (2010).

38. S. Nembrini, I. R. König, M. N. Wright, The revival of the Gini importance? *Bioinformatics* 34, 3711–3718 (2018).

39. R. Diaz-Uriarte, S. A. de Andres, Gene selection and classification of microarray data using random forest. *BMC Bioinformatics* 7, 1–13 (2006).

40. J. Li, J. Siwabessy, M. Tran, Z. Huang, A. Heap, "Predicting seabed hardness using Random Forest in R" in: Data mining applications with R, Zhao Y, Cen Y, Eds. *Elsevier* p. 299–329 (2013).

41. L. Breiman, Random forests. Machine learning, 45, 5-32 (2001).

42. M. K. Lau, DTK: Dunnett-Tukey-Kramer pairwise multiple comparison test adjusted for unequal variances and unequal sample sizes. R package version 3.5 (2013).

43. D. Barry, J. A. Hartigan, A Bayesian analysis for change point problems. *J. Am. Stat. Assoc.* 88, 309–319 (1993).

44. C. Erdman, J. W. Emerson, bcp: an R Package for performing a Bayesian analysis of change point problems. *J. Stat. Softw*. 23, 1–13 (2007).

Supporting Information

S1 Fig. Time series of broadband frequency sound pressure levels (SPLs) from 2013 until 2018. Heat maps represent temporal and spatial patterns of broadband (i.e. 1 – 40,000 Hz) frequency SPLs reflecting all biological activity and anthropogenic noise at stations (A) 9M, (B) 14M, and (C) 37M in the May River. Time is shown between noon and noon of the next day. Gaps in data = white, temperature = black line, and daylight hours = dotted line. Green dots indicate first posterior probability (PP) ⩾ 0.5 detected during springtime. At station (C) 37M first PP was not calculated for spring 2017 due to missing acoustic data.

S2 Fig. Time series of biological (i.e. files with noise removed) high frequency sound pressure levels (SPLs) from 2013 until 2018. Heat maps represent temporal and spatial patterns

of high (i.e. 7000 – 40,000 Hz) frequency SPLs reflecting snapping shrimp acoustic activity at stations (A) 9M, (B) 14M, and (C) 37M in the May River. Time is shown between noon and noon of the next day. Gaps in data = gray, files with anthropogenic noise removed = white, temperature = black line, and daylight hours = dotted line. Green dots indicate first posterior probability (PP) ⩾ 0.5 detected during springtime. At station (C) 37M first PP was not calculated for spring 2017 due to missing acoustic data.

S3 Fig. **Time series of biological (i.e. files with noise removed) low frequency sound pressure levels (SPLs) from 2013 until 2018.** Heat maps represent temporal and spatial patterns of low (i.e. 50 – 1200 Hz) frequency SPLs reflecting fish and lower portion of snapping shrimp acoustic activity at stations (A) 9M, (B) 14M, and (C) 37M in the May River. Time is shown between noon and noon of the next day. Gaps in data = gray, files with anthropogenic noise removed = white, temperature = black line, and daylight hours = dotted line. Green dots indicate first posterior probability (PP) ⩾ 0.5 detected during springtime. At station (C) 37M first PP was not calculated for spring 2017 due to missing acoustic data.

S4 Fig. Time series of biological (i.e. files with noise removed) broadband frequency sound pressure levels SPLs from 2013 until 2018. Heat maps represent temporal and spatial patterns of broadband (i.e. 1 – 40,000 Hz) frequency SPLs reflecting all biological activity at stations (A) 9M, (B) 14M, and (C) 37M in the May River. Time is shown between noon and noon of the next day. Gaps in data = gray, files with anthropogenic noise removed = white, temperature = black line, and daylight hours = dotted line. Green dots indicate first posterior probability (PP) ⩾ 0.5

detected during springtime. At station (C) 37M first PP was not calculated for spring 2017 due to missing acoustic data.

S5 Fig. Estimated mean high frequency sound pressure level (SPL) with posterior probability (PP) of change at station 9M. Posterior probability of change during springtime (light blue line) with corresponding estimated mean high (7000 – 40,000 Hz) frequency SPL (dark blue line) and corresponding water temperature (red line) in years (A) 2013, (B) 2014, (C) 2015, (D) 2016, (E) 2017, and (F) 2018. Stars indicate first positive (i.e. detected change in estimated mean SPL due to an increase not a decrease in SPL values) PP ⩾ 0.5.

S6 Fig. Estimated mean low frequency sound pressure level with posterior probability (PP) of change at station 9M. Probability of change during springtime (light blue line) with corresponding estimated mean low (50 – 1200 Hz) frequency SPL (dark blue line) and corresponding water temperature (red line) in years (A) 2013, (B) 2014, (C) 2015, (D) 2016, (E) 2017, and (F) 2018. Stars indicate first positive (i.e. detected change in estimated mean SPL due to an increase not a decrease in SPL values) PP ⩾ 0.5.

S7 Fig. Estimated mean broadband frequency sound pressure level with posterior probability (PP) of change at station 9M. Probability of change during springtime (dark blue line) with corresponding estimated mean broadband (1 – 40,000 Hz) frequency SPL (light blue line) and corresponding water temperature (red line) in years (A) 2013, (B) 2014, (C) 2015, (D) 2016, (E) 2017, and (F) 2018. Stars indicate first positive (i.e. detected change in estimated mean SPL due to an increase not a decrease in SPL values) PP ⩾ 0.5.

S8 Fig. Estimated mean high frequency sound pressure level with posterior probability (PP) of change at station 14M. Probability of change during springtime (light blue line) with corresponding estimated mean high (7000 – 40,000 Hz) frequency SPL (dark blue line) and corresponding water temperature (red line) in years (A) 2013, (B) 2014, (C) 2015, (D) 2016, (E) 2017, and (F) 2018. Stars indicate first positive (i.e. detected change in estimated mean SPL due to an increase not a decrease in SPL values) PP $\geqslant 0.5$.

S9 Fig. Estimated mean low frequency sound pressure level with posterior probability (PP) of change at station 14M. Probability of change during springtime (light blue line) with corresponding estimated mean low (50 – 1200 Hz) frequency SPL (dark blue line) and corresponding water temperature (red line) in years (A) 2013, (B) 2014, (C) 2015, (D) 2016, (E) 2017, and (F) 2018. Stars indicate first positive (i.e. detected change in estimated mean SPL due to an increase not a decrease in SPL values) PP $\geqslant 0.5$.

S10 Fig. Estimated mean broadband frequency sound pressure level with posterior probability (PP) of change at station 14M. Probability of change during springtime (light blue line) with corresponding estimated mean (1 – 40,000 Hz) broadband frequency SPL (dark blue line) and corresponding water temperature (red line) in years (A) 2013, (B) 2014, (C) 2015, (D) 2016, (E) 2017, and (F) 2018. Stars indicate first positive (i.e. detected change in estimated mean SPL due to an increase not a decrease in SPL values) PP $\geqslant 0.5$.

S11 Fig. Estimated mean high frequency sound pressure level with posterior probability (PP) of change at station 37M. Probability of change during springtime (light blue line) with corresponding estimated mean high (7000 – 40,000 Hz) frequency SPL (dark blue line) and corresponding water temperature (red line) in years (A) 2013, (B) 2014, (C) 2015, (D) 2016, (E) 2017, and (F) 2018. Stars indicate first positive (i.e. detected change in estimated mean SPL due to an increase not a decrease in SPL values) PP $\geqslant$ 0.5. Gray box = no data.

S12 Fig. Estimated mean low frequency sound pressure level with posterior probability (PP) of change at station 37M. Probability of change during springtime (light blue line) with corresponding estimated mean low (50 – 1200 Hz) frequency SPL (dark blue line) and corresponding water temperature (red line) in years (A) 2013, (B) 2014, (C) 2015, (D) 2016, (E) 2017, and (F) 2018. Stars indicate first positive (i.e. detected change in estimated mean SPL due to an increase not a decrease in SPL values) PP $\geqslant$ 0.5. Gray box = no data.

S13 Fig. Estimated mean broadband frequency sound pressure level with posterior probability (PP) of change at station 37M. Probability of change during springtime (light blue line) with corresponding estimated mean broadband (1 – 40,000 Hz) frequency SPL (dark blue line) and corresponding water temperature (red line) in years (A) 2013, (B) 2014, (C) 2015, (D) 2016, (E) 2017, and (F) 2018. Stars indicate first positive (i.e. detected change in estimated mean SPL due to an increase not a decrease in SPL values) PP $\geqslant$ 0.5. Gray box = no data.

S14 Fig. Time series of biological (i.e. files with noise removed) broadband frequency sound pressure levels (SPLs), species richness, and abundance from 2016 until 2018. Heat maps

represent temporal and spatial patterns of broadband (1 – 40,000 Hz) frequency SPLs with corresponding species richness (black line), species abundance (blue dotted line), and temperature (red line) at stations (A) 9M, (B) 14M, and (C) 37M. Files with anthropogenic noise removed = white and no data = gray box.

S15 Fig. Correlation and regression analysis of species richness and Shannon-Wiener diversity index, sound pressure levels, and temperature. Pearson's correlation between monthly averages of species richness and monthly averages of sound pressure levels (SPLs) in (A) high (7000 – 40,000 Hz), (B) low (50 – 1200 Hz), and (C) broadband (1 – 40,000 Hz) frequency ranges. Pearson's correlation (r) between monthly averages of Shannon-Wiener diversity index and monthly averages SPLs in (E) high (7000 – 40,000 Hz), (F) low (50 – 1200 Hz), and (G) broadband (1 – 40,000 Hz) frequency ranges. Linear regression between monthly averages of temperature as the independent variable and monthly averages of (D) species richness and (H) Shannon-Wiener diversity index as dependent variables between 2016 and 2018 at all stations combined. For correlations N = 86 and for regression N = 90.

S16 Fig. Correlation and regression analysis of species abundance, sound pressure levels, and temperature. Pearson's correlation between monthly averages of species richness and monthly averages of sound pressure levels (SPLs) in (A) high (7000 – 40,000 Hz), (B) low (50 – 1200 Hz), and (C) broadband (1 – 40,000 Hz) frequency ranges. Linear regression between monthly averages of temperature as the independent variable and monthly averages of (D) species abundance between 2016 and 2018 at all stations combined. For correlations N = 86 and for regression N = 90.

S1 Table. **Significance of specific variables on sound pressure levels**. Results of the Boruta, a wrapper algorithm based on random forest, that tested the significance of specific variables on high (7000 - 40000 Hz), low (50 - 1200 Hz), and broadband (1 - 40000 Hz) frequency sound pressure levels (SPLs). Decision was confirmed important at $p < 0.01$; N = 130,679.

S2 Table. Significance of specific variables on sound pressure levels. Results of the of the Boruta, a wrapper algorithm based on random forest, that tested the significance of specific variables on high (7000 - 40000 Hz), low (50 - 1200 Hz), and broadband (1 - 40000 Hz) frequency sound pressure levels (SPLs). Files that contained anthropogenic noise were removed from analysis. Decision was confirmed important at $p < 0.01$; N = 130,679.

S3 Table. **Results of first positive posterior probability of change (PP).** Year, station, mean spring water temperature, date, and day of year of first PP of change ≥ 0.5 with corresponding day anomaly, and value of PP for high (7000 - 40000 Hz) frequency sound pressure levels (SPLs).

S4 Table. **Results of first positive posterior probability of change (PP).** Year, station, mean spring water temperature, date, and day of year of first PP of change ≥ 0.5 with corresponding day anomaly, and value of PP for low (50 - 1200 Hz) frequency sound pressure levels (SPLs).

S5 Table. List of species that were caught and quantified during seining conducted one or two times per month in close proximity to passive acoustic stations in the May River, SC.

S6 Table. **Locations of National Oceanic and Atmospheric Administration (NOAA) weather stations located close to the May River, SC.** Stations were used to obtain rainfall data for each day. Data were obtained from: https://www.ncdc.noaa.gov/cdo-web/search.

Click here to download Figure Fig.1.tif

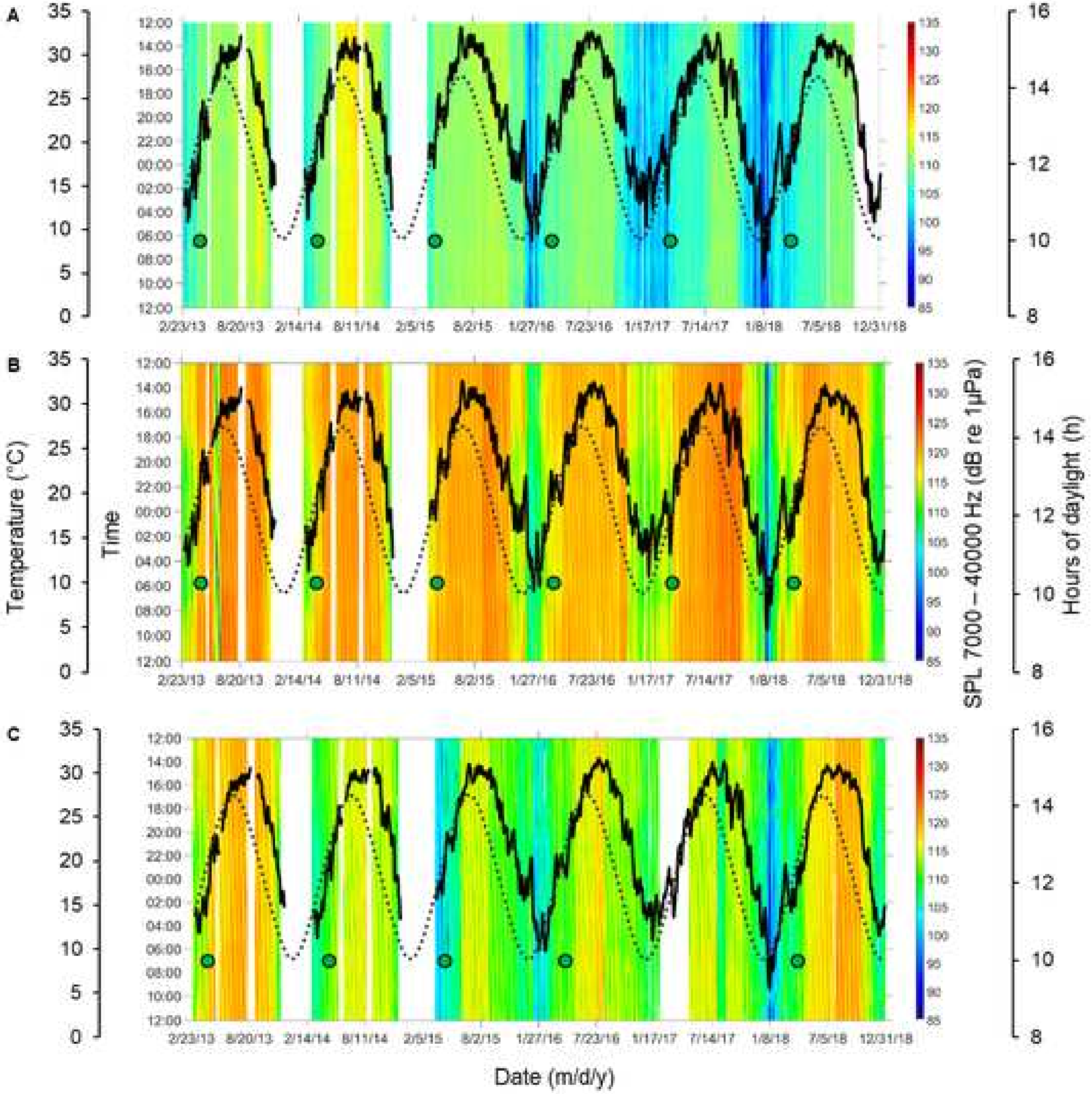

Fig 2 Click here to download Figure Fig.2.tif

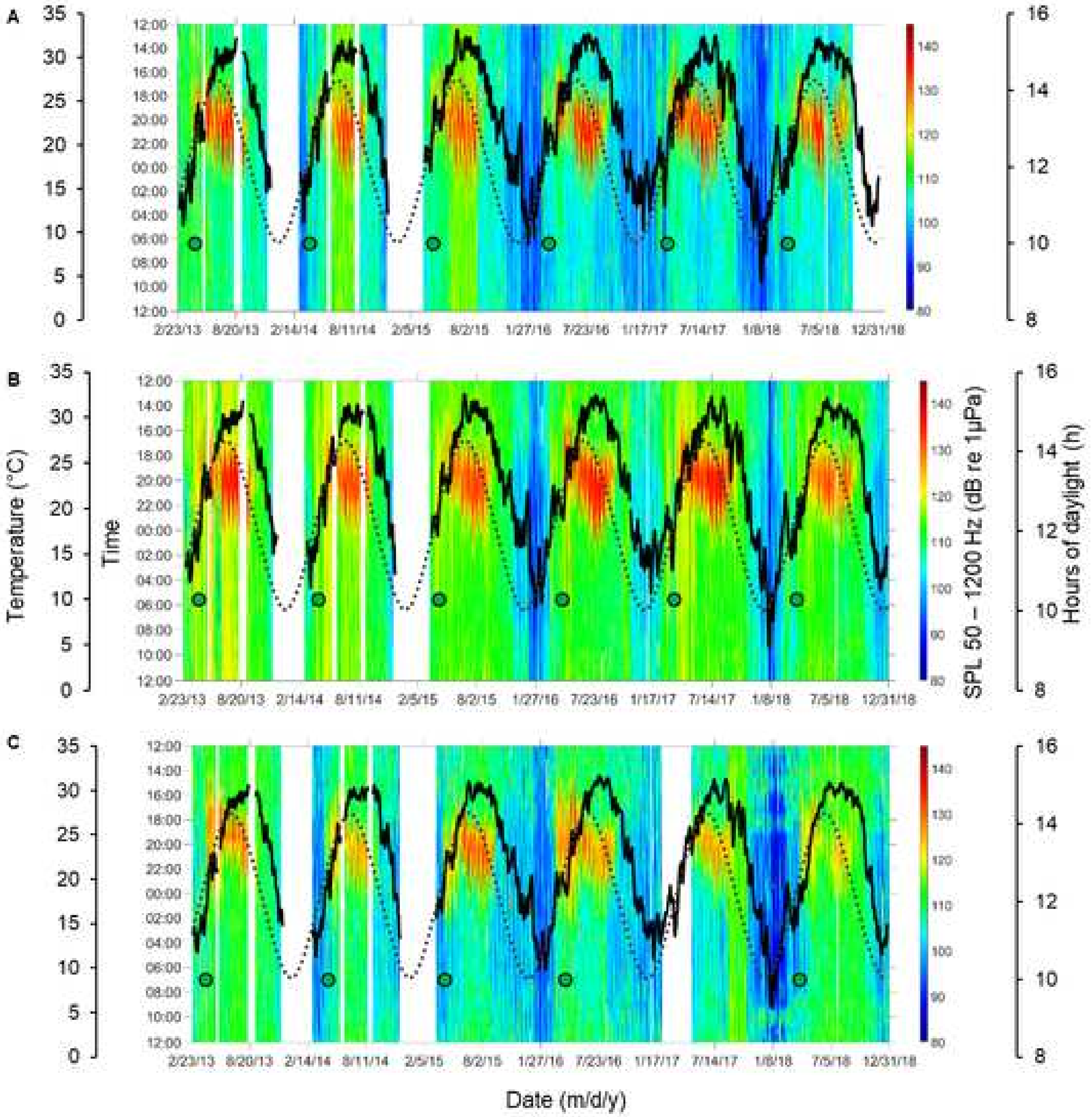

Fig 3

Click here to download Figure Fig.3.tif

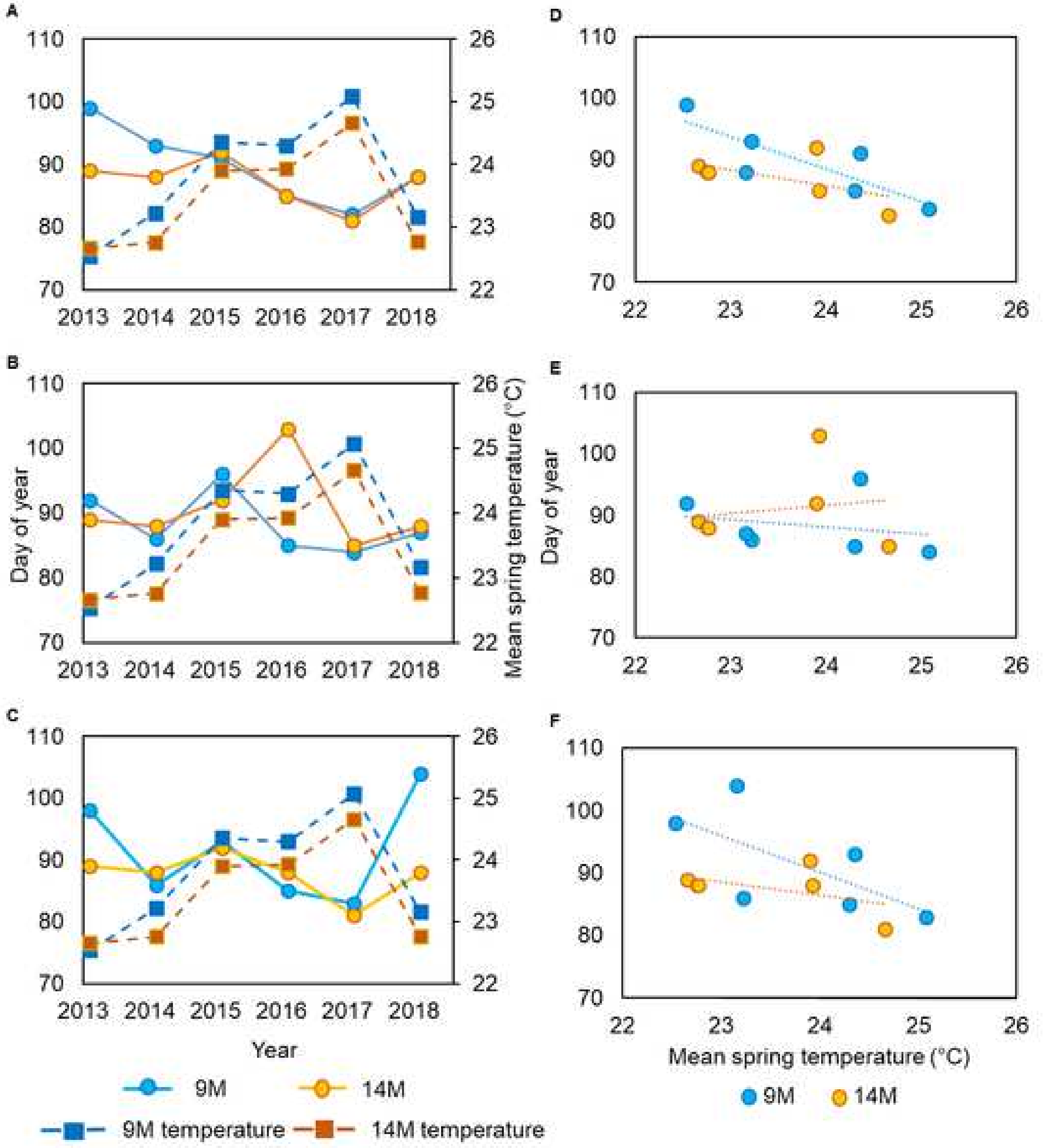

Fig 4

Click here to download Figure Fig.4.tif

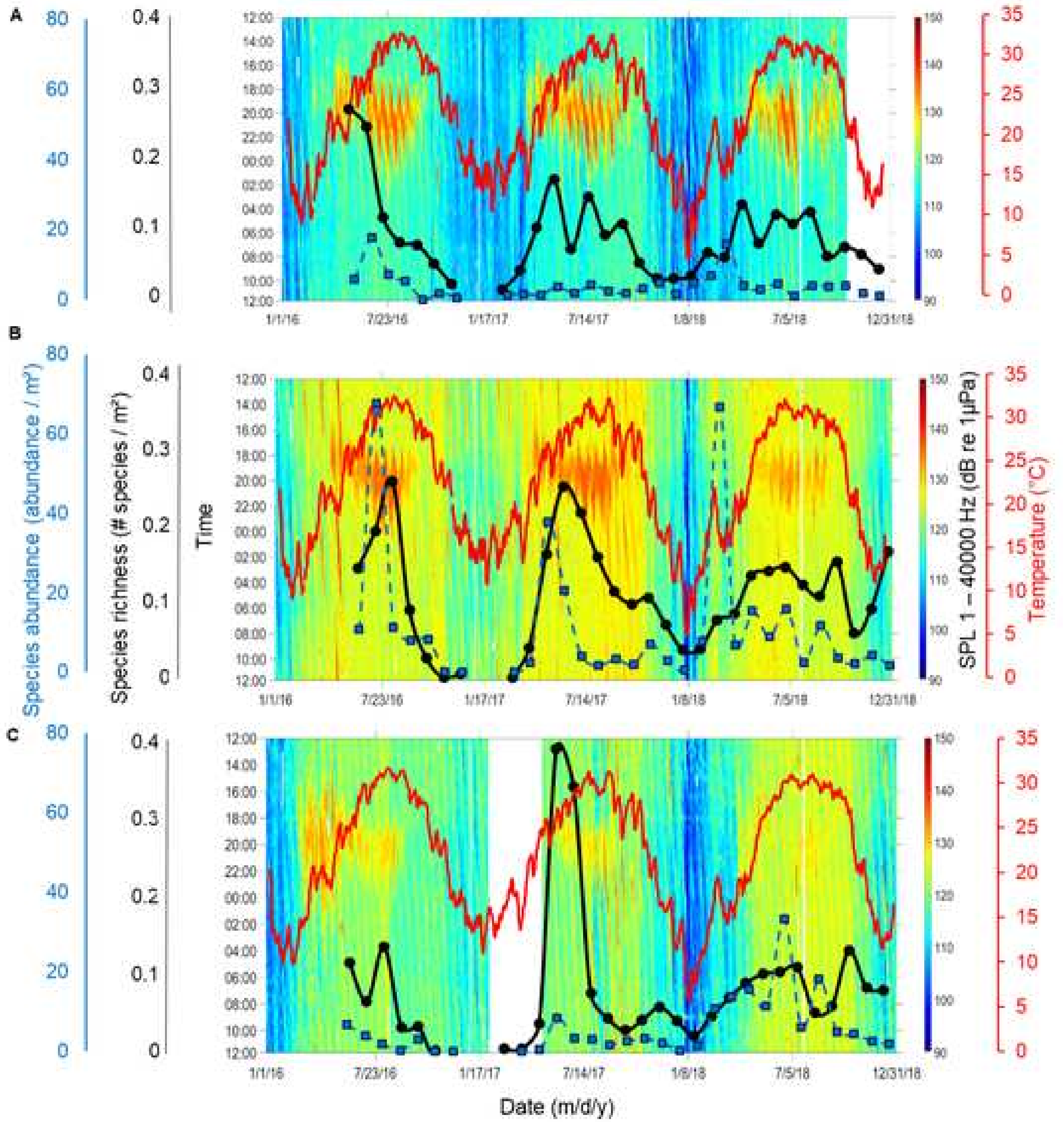

Click here to download Figure Fig.5.tif

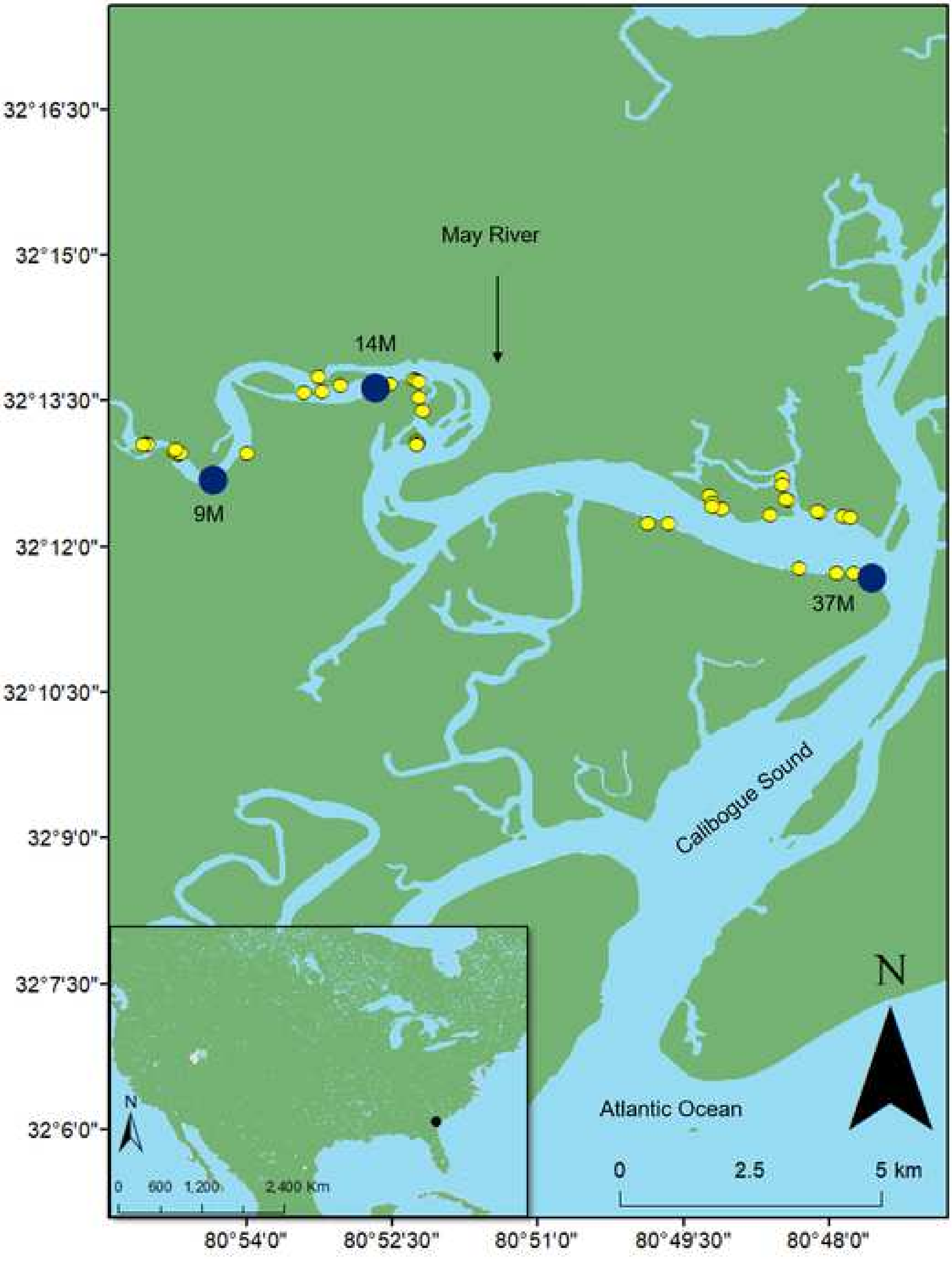

S1 Table

Click here to access/download
Supporting Information
S1 Table.xlsx

S2 Table

Click here to access/download
Supporting Information
S2 Table.xlsx

S3 Table

Click here to access/download
Supporting Information
S3 Table.xlsx

S4 Table

Click here to access/download
Supporting Information
S4 Table.xlsx

S5 Table

Click here to access/download
Supporting Information
S5 Table.xlsx

S6 Table

Click here to access/download
Supporting Information
S6 Table.xlsx

S1 Fig

Click here to access/download
Supporting Information
S1 Fig.tif

S2 Fig

Click here to access/download
Supporting Information
S2 Fig.tif

S3 Fig

Click here to access/download
Supporting Information
S3 Fig.tif

S4 Fig

Click here to access/download
Supporting Information
S4 Fig.tif

S5 Fig

Click here to access/download
Supporting Information
S5 Fig.tif

S6 Fig

Click here to access/download
Supporting Information
S6 Fig.tif

S7 Fig

Click here to access/download
Supporting Information
S7 Fig.tif

S8 Fig

Click here to access/download
Supporting Information
S8 Fig.tif

S9 Fig

Click here to access/download
Supporting Information
S9 Fig.tif

S10 Fig

Click here to access/download
Supporting Information
S10 Fig.tif

S11 Fig

Click here to access/download
Supporting Information
S11 Fig.tif

S12 Fig

Click here to access/download
Supporting Information
S12 Fig.tif

S13 Fig

Click here to access/download
Supporting Information
S13 Fig.tif

S14 Fig

Click here to access/download
Supporting Information
S14 Fig.tif

S15 Fig

Click here to access/download
Supporting Information
S15 Fig.tif

Click here to access/download
Supporting Information
S16 Fig.tif
