# Supplementary material for: What’s all that racket! Soundscapes, phenology, and biodiversity in estuaries
Source: PLoS One. 2020 Sep 3;15(9):e0236874. doi: 10.1371/journal.pone.0236874 (PMC7470342; doi:10.1371/journal.pone.0236874)
Supplement: Attachment — Submitted filename: PLoS editorial comment_Soundscapes in estuaries.docx [file pone.0236874.s026.docx]

**PLoS editorial comment_Soundscapes in Estuaries**

Line 25: Should be ‘demonstrates’

Line 42: Don’t think there should be a hyphen in knowledge gap

Line 50: Don’t capitalise ‘southeast’

Line 51: Genera should be italicised

Line 57: Spell out ‘South Carolina’ and introduce the abbreviation SC; also be consistent and use ‘USA’ as at line 50

Line 80: Should be ‘S2 Table’

Line 124: Should this be ‘S3 Table; S3 and S5 Fig’? I assume that is what the authors mean.

Line 166: A bit awkward as written. I would write ‘S15 Fig A-C, E-H and S 16 Fig A-C)

Line 167: As above

Line 170: As above

Line 174: The abbreviation for SPLs has already been introduced and does not need to be introduced again here so just write SPLs

Line 192: I think this should be a new paragraph.

Line 193: Delete the family names here as they are given in the introduction and also below on lines 199-200

Line 196: Should be ‘also have the potential’

Line 232: Use the established abbreviation SC

Line 251: Should be S1 Fig

Line 299. Full stop after SPL and write the next sentence ‘In the first set of models we included temperature as a factor, in the second set we used day length and in the third set we used rainfall.’

Line 308: Don’t introduce the abbreviation BCP as it is not used 3 times again in the paper, which is Plos formatting.

Line 324: Should be MATLAB to be consistent with how it is previously written

Line 332: Same as above
