# Supplementary material for: What’s all that racket! Soundscapes, phenology, and biodiversity in estuaries
Source: PLoS One. 2020 Sep 3;15(9):e0236874. doi: 10.1371/journal.pone.0236874 (PMC7470342; doi:10.1371/journal.pone.0236874)
Supplement: Attachment — Submitted filename: Response to Reviewers_052920_ewm.docx [file pone.0236874.s027.docx]

May 29^th^, 2020

Dear Dr. Patterson,

Enclosed please find a response to reviewers and our revised manuscript “What’s all that racket! Soundscapes, phenology, and biodiversity in estuaries.” We thank you and the reviewers for providing valuable comments and suggestions that have improved the manuscript. The reviewers’ comments are in bold and our response is in regular text. We refer to the line number changes in the track changes document. We hope the paper is now acceptable for publication. Again, thank you for your time with this publication.

Sincerely,

Eric W. Montie, M.S., Ph.D.

Marine Sensory and Neurobiology Lab

Department of Natural Sciences

University of South Carolina Beaufort

One University Boulevard

Bluffton, SC 29910, USA

Office: 843-208-8107

PONE-D-20-03292
What’s all that racket! Soundscapes, phenology, and biodiversity in estuaries
PLOS ONE

Dear Dr. Montie,

Thank you for submitting your manuscript to PLOS ONE. After careful consideration, we feel that it has merit but does not fully meet PLOS ONE’s publication criteria as it currently stands. Therefore, we invite you to submit a revised version of the manuscript that addresses the points raised during the review process. I found this to be an interesting and well written study and both reviewers agreed. However, both reviewers have suggested some relatively minor changes and clarifications to the manuscript and I have provided some minor editorial comment that need to be addressed. I encourage the authors to consider all the comments provided when making their revisions. We would appreciate receiving your revised manuscript by May 16 2020 11:59PM. When you are ready to submit your revision, log on to https://www.editorialmanager.com/pone/ and select the 'Submissions Needing Revision' folder to locate your manuscript file.
 If you would like to make changes to your financial disclosure, please include your updated statement in your cover letter. To enhance the reproducibility of your results, we recommend that if applicable you deposit your laboratory protocols in protocols.io, where a protocol can be assigned its own identifier (DOI) such that it can be cited independently in the future. For instructions see: <http://journals.plos.org/plosone/s/submission-guidelines#loc-laboratory-protocols>

Please include the following items when submitting your revised manuscript:

- A rebuttal letter that responds to each point raised by the academic editor and reviewer(s). This letter should be uploaded as separate file and labeled 'Response to Reviewers'.
- A marked-up copy of your manuscript that highlights changes made to the original version. This file should be uploaded as separate file and labeled 'Revised Manuscript with Track Changes'.
- An unmarked version of your revised paper without tracked changes. This file should be uploaded as separate file and labeled 'Manuscript'.

 Please note while forming your response, if your article is accepted, you may have the opportunity to make the peer review history publicly available. The record will include editor decision letters (with reviews) and your responses to reviewer comments. If eligible, we will contact you to opt in or out.

 We look forward to receiving your revised manuscript.

Kind regards,

Heather M. Patterson, Ph.D.
Academic Editor
PLOS ONE

Journal Requirements:

When submitting your revision, we need you to address these additional requirements:

**1. Please ensure that your manuscript meets PLOS ONE's style requirements, including those for file naming. The PLOS ONE style templates can be found at** http://www.plosone.org/attachments/PLOSOne_formatting_sample_main_body.pdf and <http://www.plosone.org/attachments/PLOSOne_formatting_sample_title_authors_affiliations.pdf>

**2. We note that you have indicated that data from this study are available upon request. PLOS only allows data to be available upon request if there are legal or ethical restrictions on sharing data publicly. For more information on unacceptable data access restrictions, please see http://journals.plos.org/plosone/s/data-availability#loc-unacceptable-data-access-restrictions.**

**In your revised cover letter, please address the following prompts:**

**a) If there are ethical or legal restrictions on sharing a de-identified data set, please explain them in detail (e.g., data contain potentially sensitive information, data are owned by a third-party organization, etc.) and who has imposed them (e.g., an ethics committee). Please also provide contact information for a data access committee, ethics committee, or other institutional body to which data requests may be sent.**

**b) If there are no restrictions, please upload the minimal anonymized data set necessary to replicate your study findings as either Supporting Information files or to a stable, public repository and provide us with the relevant URLs, DOIs, or accession numbers. For a list of acceptable repositories, please see http://journals.plos.org/plosone/s/data-availability#loc-recommended-repositories.**

**We will update your Data Availability statement on your behalf to reflect the information you provide.**

Reviewers' comments:

**Reviewer's Responses to Questions

Comments to the Author

1. Is the manuscript technically sound, and do the data support the conclusions?

The manuscript must describe a technically sound piece of scientific research with data that supports the conclusions. Experiments must have been conducted rigorously, with appropriate controls, replication, and sample sizes. The conclusions must be drawn appropriately based on the data presented.

Reviewer #1: Yes**

**2. Has the statistical analysis been performed appropriately and rigorously?

Reviewer #1: Yes

Reviewer #2: Yes**

**3. Have the authors made all data underlying the findings in their manuscript fully available?

The** [**PLOS Data policy**](http://www.plosone.org/static/policies.action#sharing) **requires authors to make all data underlying the findings described in their manuscript fully available without restriction, with rare exception (please refer to the Data Availability Statement in the manuscript PDF file). The data should be provided as part of the manuscript or its supporting information, or deposited to a public repository. For example, in addition to summary statistics, the data points behind means, medians and variance measures should be available. If there are restrictions on publicly sharing data—e.g. participant privacy or use of data from a third party—those must be specified.

Reviewer #1: Yes

Reviewer #2: Yes**

**4. Is the manuscript presented in an intelligible fashion and written in standard English?

PLOS ONE does not copyedit accepted manuscripts, so the language in submitted articles must be clear, correct, and unambiguous. Any typographical or grammatical errors should be corrected at revision, so please note any specific errors here.

Reviewer #1: Yes

Reviewer #2: Yes**

5. Review Comments to the Author

**Please use the space provided to explain your answers to the questions above. You may also include additional comments for the author, including concerns about dual publication, research ethics, or publication ethics. (Please upload your review as an attachment if it exceeds 20,000 characters)

Reviewer #1: Review – PLOS One
Manuscript number: PONE-D-20-03292
What’s all that racket! Soundscapes, phenology, and biodiversity in estuaries

General Comments:
The overall objectives of this study were to; 1. Determine temporal patterns of low, high and broadband sound pressure levels over a six-year time span, 2. Determine how certain environmental factors influence SPLs, 3. Examine the phenology of acoustic activity of snapping shrimp and sound producing fish species, and 4. Determine temporal patterns of species diversity and abundance and examine how these indices correlate with the soundscape. This study provides a valuable addition to the growing underwater soundscape field, especially while adding the fairly novel addition of phenology.
Overall, I found this manuscript to be very well written, and an interesting and well-planned investigation. I applaud the 6-year time series in acoustic data collection and effort going into this sampling!
The authors need to discuss some potentially complicating factors such as abiotics,** **water depth and propagation distances etc. (see specific comments). These factors should be touched upon in the discussion.**

Thank you. As suggested, we discussed potentially complicating abiotic factors such as water depth, and propagation distances by adding this section in the discussion (lines 211-222): “Soundscapes and limitations. In addition to sounds of biological origin (e.g. snapping shrimp and fish sounds) factors such as physical and anthropogenic sounds, water depth, and bottom topography may affect received sound pressure levels. Water flow, rain, wind, or wave action, unlike sounds of biological origin, occur randomly [23 -26]. These sounds are dominant in the low (200 - 2000 Hz) and high (15–20 kHz) frequency bandwidths [24]. However, during our analysis, we identified and removed acoustic files that contained sounds associated with intense water flow, rain, wind/wave action, and anthropogenic origin. In addition, this tidal estuary is subject to less wind and wave action as compared to open ocean environments. Thus, we are quite confident that the SPLs and patterns presented are of biological nature. Studies have shown that water depth and active space can affect sound propagation [25, 26]. In our studies, water depth and river width increased from the source towards the mouth and could potentially affect received SPL measurements.”

**Specific Comments:

Introduction
Line 64: Add ”,” between abundance & and.**

Done. **Methods**

**Lines 237 – 238: Sound propagation and potential listening distance can vary between these depths. Would be good to acknowledge and briefly discuss these complexities.**

Thank you. Studies have shown that water depth and active space can affect sound propagation [25, 26]. In our studies, water depth and river width increased from the source towards the mouth and could potentially affect received sound pressure level measurements. We acknowledged this limitation in the discussion. Please see lines 211-222.  **Lines 253-254: In the future, also consider presenting medians, especially when capturing/including anthropogenic sound sources and in some cases loud chorusing events. Root mean square SPL can be more sensitive to changes in the right tail of the probability distribution, e.g., higher noise levels, therefore the magnitude of change would be larger.**

Thank you. We will consider including medians in the feature work. **Lines 258 – 262: Revise wording for clarity. Currently it reads as though you only subsampled the data for anthropogenic sounds, and therefore removed only a subset of this? Then you state you created heatmaps representing only biological sounds – this would not be the case if you subsampled only. Or did you only graph the subsampled data?**

Thank you. Please see the methods section, which we clarified (lines 322-349).

**When noting representing biological sounds, what about abiotic sounds? I am guessing these are commonly occurring in these habitats, e.g. flow noise, wind and waves acting on the surface. Some of which would have been removed when filtering to 50 Hz, but not during broadband measurements. This should be addressed – potentially in methods or discussion.**

Please see the revised discussion section (lines 211-222) and methods section (lines 322-349)

**Results**

**Line 83: [20] Referencing Material and Methods section is somewhat confusing.
What exactly are you referencing here? If not anther study, leave out of bibliography. If referencing this current studies method, say so.**

Thank you. Done. **Line 86: Reference to supplementary figures S2-S4. How are these related? As these graphs are with the anthropogenic noise removed. Revise for clarity.**

Thank you. Figures S2-S4 represent patterns of sound pressure levels with physical sounds and anthropogenic noise removed. The reason why we removed these files was to illustrate the patterns of biological sound. In addition, removing files that contained anthropogenic noise allowed us to estimate contribution of noise to the soundscape at each station. We clarified this approach in the methods and discussion as discussed above.

**Lines 215 – 218: For a broader species view consider referencing early study showing habitat specific sound cues in promoting settlement in crustaceans:
•** **Stanley, J. A., Radford, C. A., Jeffs, A. G. Location, location, location – finding a suitable home in amongst the noise. Proceedings of the Royal Society B-Biological Sciences 279:1742, 3622 - 3631.**

Thank you. We included.  **Figures
Very nice figures throughout, however, all figures within the main article are of very poor resolution. I am thinking this is just for the review manuscript to save on size, however if not, these need to be improved for better viewing as currently they are difficult to view and read in some places.**

Thank you. We will submit the figures as tifs for the final manuscript.  **Figures 1 & 2: In Figure 1 the data gaps are in white (as per figure legend), in Figure 2 they are in Grey, although the figure legend notes white. This is probably just an oversite but stay consistent for clarity. Personally, I think grey illustrates it the best.**

Both figures 1 & 2 have gaps in data plotted in white, and we indicated that in the figure legends.  **Figure 5: might be good if you could indicate what the blue and yellow icons were in a small inset of the figure for ease of viewing.**

Thank you. We added insert with legend indicating blue and yellow icons. Please see Fig. 5. **Reviewer #2: This study explored the phenology and biodiversity of a subtidal river using underwater acoustic recordings. The authors collected six-years of data, which demonstrated an increase in biotic sounds in years with warmer springs. This is a very interesting result, and is particularly relevant given global climate change.

However, the manuscript requires some clarifications. I have two key concerns:

Firstly, in the Methods it says that a subset of the data were manually reviewed to remove files containing anthropogenic noise - however, the Results then seem to alter between using the full dataset (i.e. containing anthropogenic noise) and the subset data (i.e. excluding anthropogenic noise). It would be beneficial to clarify throughout when you are using which dataset. Naming them could help with this.

Secondly, the subset data is split into low frequency (50 - 1200 Hz) and high frequency (7000 - 40,000 Hz). It then appears to be assumed that all sounds in the low-frequency category are caused by fish and those in the high-frequency category are caused by snapping shrimp. This is likely broadly true - but the assumption is not explicitly stated anywhere in the manuscript. Although the anthropogenic noise has been removed, there are still other sound sources that could be contributing to noise levels in these low- and high-frequency categories. For example, abiotic sounds (i.e. rain, wind, currents, bubbles, etc) can considerably alter ambient noise levels. See Marley et al (2016) Underwater sound in an urban estuarine river (DOI 10.1007/s40857-015-0038-z); here I found that wind strongly influenced the local soundscape.** **I appreciate that abiotic sounds can be difficult (impossible?) to remove; but as they are natural sounds, there is still the risk that they could be correlated with temperature (e.g. weather patterns, higher flow rates in the river, etc) and thus influence results. Rather than redoing any data review or analysis, I would like to see this assumption fully addressed in the Methods section.

Apart from these two key concerns, my comments are relatively minor suggestions. I have attached an annotated PDF of specific comments. In particular, I think it would be worth** **developing the Discussion further to highlight the relevance of this research in a changing climate. This is of particular importance given your own point in the Introduction - phenology is relatively overlooked in marine systems compared to terrestrial ones. So please do take the opportunity to elaborate further in the Discussion.

I look forward to seeing the final publication!

All the best,

Sarah Marley**

Thanks Sarah for the kind and thorough review. Many of your suggestions were similar to Reviewer 1. Please see the changes below:

#1 Comment and Response: Please see the methods section, which we clarified (lines 322-349). We determined the root mean square (rms) SPL for the entire data set (i.e. each 2 min wav file every 20 min; 392,106 files for all 3 stations) for high (i.e. 7000 – 40,000 Hz), low (i.e. 50 – 1200 Hz), and broadband (i.e. 1 – 40,000 Hz) frequencies using custom scripts created in MATLAB R2017b (MathWorks, Inc., Natick, MA, USA). We chose these ranges based on previous studies that revealed specific call frequencies for black drum (70 – 90 Hz), silver perch (1000 – 1280 Hz), oyster toadfish (190 – 200 Hz), spotted seatrout (200 – 270 Hz), red drum (120 – 160 Hz), and snapping shrimp (50 – 40 kHz) [20]. In the May River estuary, previous studies have discovered that the soundscape is composed mainly of biological sounds (i.e. snapping shrimp, fish, and bottlenose dolphins), physical sounds (i.e. wave, wind, water flow, and rain), and anthropogenic noise (i.e. recreational boat) [18]. Broadband frequency SPL values reflected biological sounds (i.e. snapping shrimp, fish, and marine mammals), physical sounds (i.e. wave, wind, water flow, and rain), and anthropogenic noise (i.e. recreational boats) [18]. Low frequency SPLs included fish calls, the lower bandwidth of snapping shrimp snaps, bottlenose dolphin vocalizations (which were few and random), physical sounds, and anthropogenic noise [18]. High frequency SPLs included snapping shrimp snaps, high frequency vocalizations of bottlenose dolphins, physical sounds, and anthropogenic noise [18].

In order to decipher biological patterns from physicals sounds and noise, we subsampled the data set and manually analyzed files recorded on the hour (i.e. 130,702 files for all 3 stations). We flagged the files that contained biological sounds (i.e. snapping shrimp, fish, and bottlenose dolphin), physical sounds (i.e. wave, wind, water flow, and rain), and anthropogenic noise (i.e. recreational boats) [18]. Then, we removed the files that contained physical sounds and anthropogenic noise from the subsampled data set and reanalyzed high, low, and broadband SPLs. This approach ensured that the patterns observed where of biological nature. We created heat maps in MATLAB R2017b using the entire data set representing all SPL values (i.e. 2 min on the hour including sounds of biological, physical, and anthropogenic origin) and the subsampled data set (i.e. 2 min on the hour including only sounds of biological origin). These SPL values were plotted versus date and time at each acoustic station (i.e. 9M, 14M, and 37M) with corresponding water temperature and daylight hours.

#2 Comment and Response: As suggested, we discussed potentially complicating abiotic factors such as water depth, and propagation distances by adding this section in the discussion (lines 211-222): “Soundscapes and limitations. In addition to sounds of biological origin (e.g. snapping shrimp and fish sounds) factors such as physical and anthropogenic sounds, water depth, and bottom topography may affect received sound pressure levels. Water flow, rain, wind, or wave action, unlike sounds of biological origin, occur randomly [23 -26]. These sounds are dominant in the low (200 - 2000 Hz) and high (15–20 kHz) frequency bandwidths [24]. However, during our analysis, we identified and removed acoustic files that contained sounds associated with intense water flow, rain, wind/wave action, and anthropogenic origin. In addition, this tidal estuary is subject to less wind and wave action as compared to open ocean environments. Thus, we are quite confident that the SPLs and patterns presented are of biological nature. Studies have shown that water depth and active space can affect sound propagation [25, 26]. In our studies, water depth and river width increased from the source towards the mouth and could potentially affect received SPL measurements.”

#3 Comment and Response: We took your advice and developed the Discussion further to highlight the relevance of this research in a changing climate and the lack of phenology studies in marine environments (see lines 239-257)

**Line 52: This is rather vague - can you give some more specific examples?**

Thank you, we added specific examples to the manuscript, see lines: 52 -59.

**Line 69: Is this what you expected? If so, is it part of the justification for choosing these ranges in the first place?**

We added this information in the methods section and addressed above (see lines 322-349).

**Line 71: And anthropogenic origin? Or were these the files that had been manually reviewed to remove anthropogenic sounds? i.e. are these ALL files or a subset?**

See our response above and lines 322-349.

**Line 77: This sounds like the subset data ... so presumably Line 71 above was referring to all the data (inc anthro noise)?**

See our response above and lines 322-349.

**Line 85: Any comment on the type of anthropogenic noise? Presumably vessel traffic?**

Yes, in the May River, recreational boats are the primary source of anthropogenic noise. We clarified in line 93.

**Line 94: How was this quantified? Snaps per minute? Automatic detector? Manual review? SPL in a particular frequency band?**

Thank you. We added this information to lines 105-106. Snapping shrimp acoustic activity (i.e. measured as high 7000 – 40,000 Hz frequency SPLs) was higher during the day, low tide, and new moon as compared to the night, high tide, and full moon (p < 0.05).

**Line 98: Again, it is very unclear from the terminology used when this includes all files vs anthropogenic-removed files.**

Thank you. We clarified in the manuscript that these analyses were performed on the data set that excluded physical sounds and anthropogenic noise. Please see lines 86-87 and 98-100.

**Line 120: Is this how you quantified snapping shrimp acoustic activity as being higher at certain times (i.e. Line 94)? I'm concerned that there may be other sound sources occurring in these HF/LF SPL categories that could be misleading. Yes, the files with anthropogenic noise have been removed (although it is still unclear when these data are being used or not), but what about abiotic sounds (e.g. precipitation, wind)? Abiotic sources can considerably raise the ambient noise levels. I'm sure that this has been considered - but this needs to be explicitly stated in the Methods.**

Thank you. See our response above and lines 322-349.

**Line 160: Any known soniferous species?**

Yes, we revised Table S5 to include soniferous organisms and adjusted the text in the results section to reflect this change (see lines 173-175). However, many of the sound-producing fish species were young-of-the-year, so we are not sure about their ability to produce sound. We did collect juvenile Atlantic croaker, silver perch, spotted seatrout, red drum, black drum, and weakfish.

**Line 168: So at colder water temperatures, you have a lot of a few species - and warmer temperatures you have a few of many species? Or is this species-specific abundance rather than overall abundance? This might have been clearer in the Supplementary Figures, but unfortunately I'm struggling to download them! Does this then evidence that fish are indeed calling less at colder temperatures, rather than just moving away to different areas when it's cooler. Which species of fish remain at lower temps? Are they soniferous?**

We detected temporal patterns in species richness, the Shannon-Wiener diversity index, and abundance of invertebrates and fish species. We found lower species diversity and abundance during wintertime (i.e. cooler season) and higher species diversity and abundance during spring and summertime (i.e. warmer seasons). We clarified these findings in the results (see lines 177 -179).

As we stated previously, many of the sound-producing fish species collected in seines were young-of-the-year, and the ability of this life stage to produce sound is questionable. In the May River estuary, adult male oyster toadfish, silver perch, spotted seatrout, and red drum are the major sound-producing species that produce courtship calls/choruses associated with spawning. These fish contribute predominantly to the sound pressure levels in the low frequency bandwidth. Oyster toadfish, silver perch, and spotted seatrout are residents in estuaries year-round, while adult red drum may move offshore in the colder winter months. In the May River estuary, none of these fish (oyster toadfish, black drum, silver perch, spotted seatrout, and red drum) produces sound in the cold-winter months from November to February. We clarified these patterns in the text but placed in the Discussion section (see lines 260-268).

**Line 197 -200:** **Do they definitely decrease calling? Or could the fish be leaving the area to go elsewhere when the temperature cools? I think this should be highlighted more in your 'Soundscape and Biodiversity' section of the Results.**

Please see our comments in the prior response.

**Line 214: Were any of your recording sites near such features?**

The May River is a salt marsh estuary bordered by extensive patches of smooth cordgrass *Spartina alterniflora* and oyster reefs comprised of the eastern oyster *Crassostrea virginica*. Passive acoustic recorders were placed on the bottom, close to the sides of the estuary, where live and dead oyster patches were common. We added this information in the discussion. Please see lines 279-283.

**Line 227: I feel like this point could have been explored in more detail in this Discussion. There is no mention of climate change or what changing temperatures could mean for phenology / biodiversity of marine systems. As noted in the Introduction, this has been explored in detail for many terrestrial systems / species, but the marine side has been vastly overlooked. Therefore, it is worth taking the time to discuss here.**

Thank you. We elaborated more on this topic in the discussion. Please see lines 239-257.

**Line 255: What was the justification for defining low and high in this way? Why was there a cap of 1200 - 7000 Hz?**

We investigated SPLs in the high (i.e. 7000 – 40,000 Hz), low (i.e. 50 – 1200 Hz), and broadband (i.e. 1 – 40,000 Hz) frequencies. We chose these ranges based on previous studies that revealed specific call frequencies for black drum (70 – 90 Hz), silver perch (1000 – 1280 Hz), oyster toadfish (190 – 200 Hz), spotted seatrout (200 – 270 Hz), red drum (120 – 160 Hz), and snapping shrimp (50 – 40 kHz) [20]. Broadband frequency SPL values reflected biological sounds (i.e. snapping shrimp, fish, and marine mammals), physical sounds (i.e. wave, wind, water flow, and rain), and anthropogenic noise (i.e. recreational boats) [18]. Low frequency SPLs included fish calls, the lower bandwidth of snapping shrimp snaps, bottlenose dolphin vocalizations (which were few and random), physical sounds, and anthropogenic noise [18]. High frequency SPLs included snapping shrimp snaps, high frequency vocalizations of bottlenose dolphins, physical sounds, and anthropogenic noise [18]. We added this information to the manuscript. Please see lines 322-337.

**Line 260: It would be useful to know a bit more about the study area. What types of abiotic / biotic / anthropogenic noise would you expect here based on previous studies?**

In the May River estuary, the soundscape is composed mainly of biological (i.e. snapping shrimp, fish, and bottlenose dolphin), physical (i.e. wave, wind, water flow, and rain), and anthropogenic (i.e. recreational boat) sounds [18]. We added this information to the manuscript (see lines 328-331).

**Line 275: This implies there was a number of dead organisms - can you please quantify this?**

We transferred the catch into a live well, quantified the abundance of each species, and then released all the organisms at the original sampling location. Mortality of the organisms was low during the wintertime (< 5 %) and moderate during summertime (~5 to 50%). We added this information to lines 361-364.

**Line 279: Define as mean vs median?**

Thank you. We calculated the mean. We clarified in the text, line 367.

**6. PLOS authors have the option to publish the peer review history of their article (**[**what does this mean?**](https://journals.plos.org/plosone/s/editorial-and-peer-review-process#loc-peer-review-history)**). If published, this will include your full peer review and any attached files.


If you choose “no”, your identity will remain anonymous but your review may still be made public.


Do you want your identity to be public for this peer review? For information about this choice, including consent withdrawal, please see our** [**Privacy Policy**](https://www.plos.org/privacy-policy)**.

Reviewer #1: No

Reviewer #2: Yes: Sarah Marley


[NOTE: If reviewer comments were submitted as an attachment file, they will be attached to this email and accessible via the submission site. Please log into your account, locate the manuscript record, and check for the action link "View Attachments". If this link does not appear, there are no attachment files to be viewed.]

While revising your submission, please upload your figure files to the Preflight Analysis and Conversion Engine (PACE) digital diagnostic tool,** [**https://pacev2.apexcovantage.com/**](https://pacev2.apexcovantage.com/)**. PACE helps ensure that figures meet PLOS requirements. To use PACE, you must first register as a user. Registration is free. Then, login and navigate to the UPLOAD tab, where you will find detailed instructions on how to use the tool. If you encounter any issues or have any questions when using PACE, please email us at** [**figures@plos.org**](mailto:figures@plos.org)**. Please note that Supporting Information files do not need this step.**

PLoS editorial comment_Soundscapes in Estuaries

**Line 25: Should be ‘demonstrates’**

Done.

**Line 42: Don’t think there should be a hyphen in knowledge gap**

Done.

**Line 50: Don’t capitalize ‘southeast’**

Done.

**Line 51: Genera should be italicized**

Done.

**Line 57: Spell out ‘South Carolina’ and introduce the abbreviation SC; also be consistent and use ‘USA’ as at line 50**

Done.

**Line 80: Should be ‘S2 Table’**

Done.

**Line 124: Should this be ‘S3 Table; S3 and S5 Fig’? I assume that is what the authors mean.**

Yes.

**Line 166: A bit awkward as written. I would write ‘S15 Fig A-C, E-H and S 16 Fig A-C)**

Done.

**Line 167: As above**

Done.

**Line 170: As above**

Done.

**Line 174: The abbreviation for SPLs has already been introduced and does not need to be introduced again here so just write SPLs**

Done.

**Line 192: I think this should be a new paragraph.**

Done.

**Line 193: Delete the family names here as they are given in the introduction and also below on lines 199-200**

Done.

**Line 196: Should be ‘also have the potential’**

Done.

**Line 232: Use the established abbreviation SC**

Done.

**Line 251: Should be S1 Fig**

Done.

**Line 299. Full stop after SPL and write the next sentence ‘In the first set of models we included temperature as a factor, in the second set we used day length and in the third set we used rainfall.’**

Done.

**Line 308: Don’t introduce the abbreviation BCP as it is not used 3 times again in the paper, which is Plos formatting.**

Done.

**Line 324: Should be MATLAB to be consistent with how it is previously written**

Done.

**Line 332: Same as above**

Done.

*In compliance with data protection regulations, you may request that we remove your personal registration details at any time.* [*(Remove my information/details)*](https://www.editorialmanager.com/pone/login.asp?a=r)*. Please contact the publication office if you have any questions.*
